# HER3 activation contributes toward the emergence of ALK inhibitor-tolerant cells in ALK-rearranged lung cancer with mesenchymal features

**DOI:** 10.1038/s41698-021-00250-8

**Published:** 2022-01-18

**Authors:** Keiko Tanimura, Tadaaki Yamada, Koutaroh Okada, Kunihiro Nakai, Mano Horinaka, Yuki Katayama, Kenji Morimoto, Yuri Ogura, Takayuki Takeda, Shinsuke Shiotsu, Kosuke Ichikawa, Satoshi Watanabe, Yoshie Morimoto, Masahiro Iwasaku, Yoshiko Kaneko, Junji Uchino, Hirokazu Taniguchi, Kazue Yoneda, Satoaki Matoba, Toshiyuki Sakai, Hisanori Uehara, Seiji Yano, Tetsuro Kusaba, Ryohei Katayama, Koichi Takayama

**Affiliations:** 1 Department of Pulmonary Medicine, Graduate School of Medical Science, Kyoto Prefectural University of Medicine, 465 Kajii-Cho Kawaramachi-Hirokoji, Kamigyo-Ku, Kyoto 602-8566 Japan; 2 Cancer Chemotherapy Center, Japanese Foundation for Cancer Research, 3-8-31, Ariake, Koto, Tokyo 135-8550 Japan; 3 Department of Nephrology, Graduate School of Medical Science, Kyoto Prefectural University of Medicine, 465 Kajii-Cho Kawaramachi-Hirokoji, Kamigyo-Ku, Kyoto, 602-8566 Japan; 4 Department of Drug Discovery Medicine, Graduate School of Medical Science, Kyoto Prefectural University of Medicine, 465 Kajii-Cho Kawaramachi-Hirokoji, Kamigyo-Ku, Kyoto, 602-8566 Japan; 5 Department of Respiratory Medicine, Japanese Red Cross Kyoto Daini Hospital, 355-5 Haruobi-Cho, Kamigyo-Ku, Kyoto, 602-8026 Japan; 6 Department of Respiratory Medicine, Japanese Red Cross Kyoto Daiichi Hospital, 15-749, Honmachi, Higashiyama-Ku, Kyoto, 605-0981 Japan; 7 Department of Respiratory Medicine and Infectious Diseases, Niigata University Graduate School of Medical and Dental Sciences, 2-5274, Gakkocho-Dori, Niigata, 951-8514 Japan; 8 Department of Respiratory Medicine, Nagasaki University Graduate School of Biomedical Sciences, 1-12-4, Sakamoto, Nagasaki, 852-8523 Japan; 9 University of Occupational and Environmental Health, Second Department of Surgery, 1-1, Iseigaoka, Kitakyushu, Fukuoka, 807-8556 Japan; 10 Department of Cardiovascular Medicine, Graduate School of Medical Science, Kyoto Prefectural University of Medicine, 465 Kajii-Cho Kawaramachi-Hirokoji, Kamigyo-Ku, Kyoto, 602-8566 Japan; 11 Division of Pathology, Tokushima University Hospital, 2-50-1 Kuramotocho, Tokushima City, Tokushima, 770-8503 Japan; 12 Division of Medical Oncology, Cancer Research Institute, Kanazawa University, Kakuma-Machi, Kanazawa, Ishikaswa 920-1192 Japan

**Keywords:** Translational research, Non-small-cell lung cancer

## Abstract

Anaplastic lymphoma kinase-tyrosine kinase inhibitors (ALK-TKIs) have shown dramatic efficacy in patients with ALK-rearranged lung cancer; however, complete response in these patients is rare. Here, we investigated the molecular mechanisms underlying the emergence and maintenance of drug-tolerant cells in ALK-rearranged lung cancer. Cell based-assays demonstrated that HER3 activation and mesenchymal-to-epithelial transition, mediated through ZEB1 proteins, help maintain cell survival and induce the emergence of ALK-TKI-tolerant cells. Compared with ALK-TKIs alone, cotreatment with pan-HER inhibitor afatinib and ALK-TKIs prevented tumor regrowth, leading to the eradication of tumors in ALK-rearranged tumors with mesenchymal features. Moreover, pre-treatment vimentin expression in clinical specimens obtained from patients with ALK-rearranged lung cancer was associated with poor ALK-TKI treatment outcomes. These results demonstrated that HER3 activation plays a pivotal role in the emergence of ALK-TKI-tolerant cells. Furthermore, the inhibition of HER3 signals combined with ALK-TKIs dramatically improves treatment outcomes for ALK-rearranged lung cancer with mesenchymal features.

## Introduction

Lung cancer is the leading cause of cancer-related death worldwide^1^, with non-small cell lung cancer (NSCLC) accounting for approximately 85% of all lung cancer cases^2^. The treatment strategy for patients with NSCLC recently changed from a histology-based approach to a molecular-targeted approach due to the identification of targetable driver oncogenes and critical developments in molecular-targeted therapies^3,4^. In 2007, an echinoderm microtubule-associated protein-like 4 (*EML4*)-anaplastic lymphoma kinase (*ALK*) fusion gene was discovered in NSCLC^5^, and it has since been shown that approximately 3–5% of NSCLC patients harbor ALK-rearrangement and display dramatic responses to ALK-tyrosine kinase inhibitors (ALK-TKIs)^6,7^. There are various classes of ALK-TKIs; however, second-generation ALK-TKIs, alectinib and brigatinib, have demonstrated significantly longer progression-free survival (PFS) than the first generation ALK-TKI crizotinib when used as a first-line treatment for ALK-positive NSCLC patients^8–10^. Based on positive clinical trial results, alectinib and brigatinib are currently approved as a standard first-line treatment for NSCLC with *ALK* rearrangements in the US, Japan, and other countries. Recently, the third generation ALK-TKI lorlatinib was approved to treat refractory ALK-positive NSCLC patients^11^; however, as complete response to any of the classes of ALK-TKIs is rare, almost all patients with *ALK*-rearranged NSCLC inevitably acquire resistance to ALK-TKIs, including alectinib and brigatinib.

Multiple factors reportedly affect acquired resistance to ALK-TKIs, including secondary ALK-resistant mutations through G1202R, L1196M, and I1171T/N/S point mutations, *ALK* amplification, bypass signaling activations, such as EGFR, HER3, IGF-1R, cMET, or AXL, epithelial-mesenchymal transition (EMT), P-glycoprotein overexpression, and the emergence of other fusion genes, such as *NRG1* and *RET*^12,13^. While second and third-generation ALK-TKIs are effective against acquired crizotinib resistance, they have a limited clinical ability to conquer acquired resistance, with the exception of some secondary ALK resistance mutations^14,15^. Therefore, it is necessary to elucidate the mechanisms underlying the prevention of drug resistance to improve the prognosis of patients with NSCLC with ALK-rearrangement.

In recent years, increasing attention has been paid to the concept of drug tolerance, wherein small subpopulations of reversibly drug-tolerant (DT) cells develop with 100-fold resistance to anti-cancer therapeutic agents, including molecularly targeted therapies, compared with parental cells^16^. Some studies have reported molecular mechanisms related to drug tolerance in EGFR mutant NSCLC, such as IGF-1R signaling activation, lipid hydroperoxidase glutathione peroxidase 4 (GPX4) dependency, aldehyde dehydrogenase (ALDH) upregulation, and the activation of β-catenin signaling, AXL or Aurora kinase A^16–18^. However, little is known about the mechanisms that modulate ALK-TKI tolerance in DT cells from ALK-rearranged NSCLC.

In this study, we investigated the molecular mechanisms underlying the emergence and maintenance of DT cells by treating ALK-rearranged NSCLC cells with ALK inhibitors. Moreover, we attempted to develop a novel therapeutic strategy and identify a predictive biomarker for the prevention of DT cell emergence following ALK-TKI intervention, owing to the eradication of tumor activity in ALK-rearranged lung cancer.

## Results

### HER3 activation plays pivotal role in the emergence of ALK-TKI-tolerant cells

**Fig. 1:**
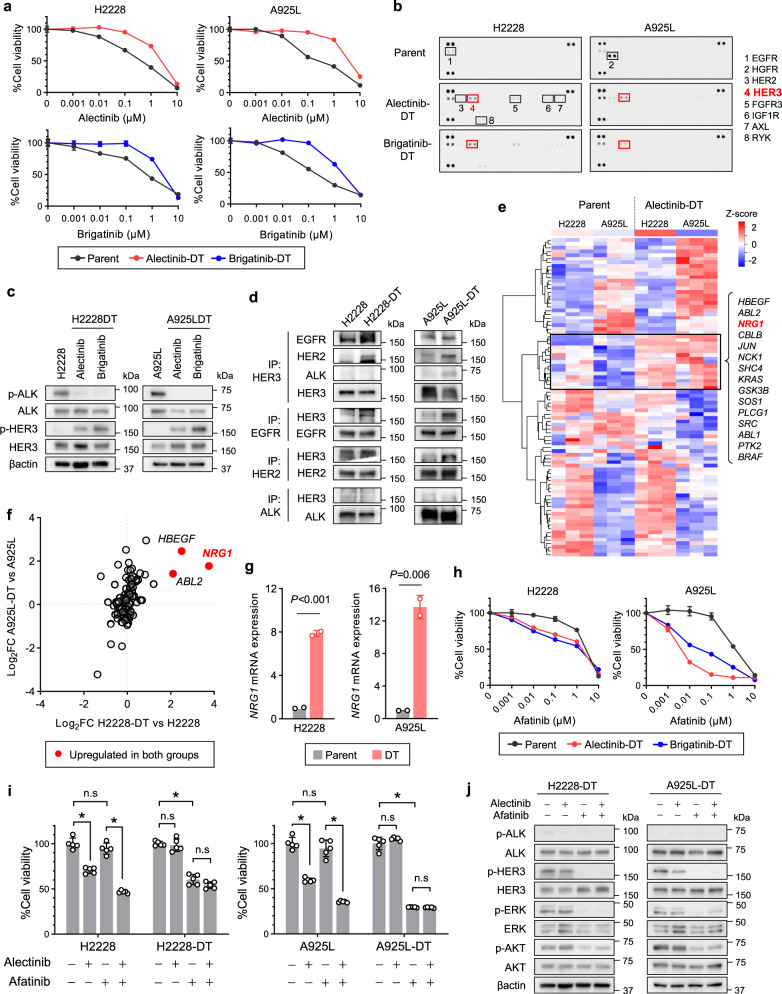
HER3 activation plays a pivotal role in the emergence of ALK-TKI-tolerant ALK-rearranged NSCLC cells. **a** H2228 and A925L parental and DT cells treated with alectinib (3 µmol/L) or brigatinib (1 µmol/L) for 9 days were incubated with the indicated concentrations of alectinib or brigatinib for 72 h. Cell viability was detected using MTT assays. **b** Human phospho-RTK array analysis of parental and DT cells treated with ALK-TKIs. Western blotting (**c**) and with immunoprecipitation (**d**) of parental and DT cells. **e** Microarray analysis of H2228 and A925L parental vs. DT cells. Heatmap and *Z*-score hierarchical clustering of differentially expressed genes associated with KEGG_ERBB_SIGNALING_PATHWAY gene set. Highlighted genes are upregulated in DT cells of both H2228 and A925L. **f** Scatterplot of genes in the KEGG_ERBB_SIGNALING_PATHWAY gene set with significant differential expression in either H2228 or A925L DT cells vs parent cells. *x*-Axis: log_2_FC of the gene between H2228-DT vs. H2228; *y*-axis: log_2_FC of the same gene between A925L-DT and A925L cells. Samples are colored according to the significance of differential expression (absolute log_2_FC > 1) in both groups. **g** qPCR of neuregulin 1 (*NRG1*) in parental and DT cells. *P*-values were calculated by unpaired *t* tests. **h** Parental and DT cells were incubated with afatinib for 72 h and **i** treated with medium only, alectinib, afatinib, or a combination for 72 h. **h**, **i** Cell viability MTT assays. *P < 0.05 (two*-*way ANOVA followed by Tukey’s test). **j** Western blotting of parental and DT cells treated with 100 nmol/L of alectinib, afatinib, or a combination. Data are represented as mean ± S.D.

DT cells are a small subpopulation of cells with reduced sensitivity to targeted drugs that are generally established within several days to weeks of exposure to the targeted drugs^16^. Herein, we isolated DT cells from H2228 and A925L cells exposed to high doses of alectinib (3 µmol/L) or brigatinib (1 µmol/L) for 9 days (Supplementary Fig. 1a). Compared to their parental cells, these DT cells were cross-resistant to several ALK-TKIs (alectinib, brigatinib, crizotinib, and lorlatinib; Fig. 1a and Supplementary Fig. 1b) and a larger population were in the G1 phase of the cell cycle, as reported previously^17^ (Supplementary Fig. 1c).

To determine the mechanisms underlying ALK-TKI resistance, we compared the expression of 49 phospho-RTKs in H2228 and A925L cells with their DT cells using a phospho-RTK antibody array. HER3 phosphorylation was higher in DT cells than in their parental cells (Fig. 1b), suggesting that ALK-TKI tolerance may at least partially result from survival signals through HER3 activation. It was also revealed that ALK-TKI-resistant DT cells displayed higher total and phosphorylated HER3 protein levels than their parental H2228 and A925L cells. Conversely, ALK phosphorylation was lower in DT cells than in the parental cells, suggesting that DT cell viability is independent of ALK (Fig. 1c). Immunoprecipitation experiments revealed that the binding of HER3 to EGFR and HER2 was enhanced in DT cells (Fig. 1d and Supplementary Fig. 1d), suggesting that DT cell survival was maintained by HER3 signaling in some ALK-rearranged NSCLC cells, presumably via interactions with EGFR or HER2.

To further identify pathways associated with DT cell characteristics, we performed transcriptome analysis between parental and DT cells using a gene expression microarray. Compared with parental cells, GSEA demonstrated increased expression of ERBB signaling pathway-dependent signature genes in DT cells (Supplementary Fig. 2a, b). Among several expressed genes associated with the ERBB pathway, neuregulin 1 (*NRG1*)—a HER3 ligand—showed the most upregulation in DT cells compared with parental cells (Fig. 1e, f). Furthermore, DT cells expressed significantly higher mRNA and protein levels of *NRG1* than parental cells, suggesting that ligand-dependent HER3 activation is important for DT cell viability (Fig. 1g and Supplementary Fig. 2c).

To examine the roles of HER3 signaling in DT cells, we used the pan-HER family tyrosine kinase inhibitor afatinib. Afatinib decreased the viability of DT cells, but not parental H2228 or A925L cells, whereas treatment with both alectinib and afatinib had no additional effect on DT cell viability compared with afatinib alone (Fig. 1h, i). Similar results were observed following treatment with anti-human HER3 neutralizing antibodies, which competitively block ligand-dependent HER3 binding (Supplementary Fig. 2d). Conversely, when treated with alectinib, the recombinant NRG1 stimulated HER3 and its downstream AKT and ERK signaling pathways, thereby attenuating alectinib sensitivity (Supplementary Fig. 2e, f). Western blotting revealed that although alectinib did not inhibit HER3, ERK, or AKT phosphorylation in H2228 and A925L DT cells, afatinib inhibited HER3 phosphorylation, and thus suppressed AKT and ERK phosphorylation (Fig. 1j). Together, these results indicate that HER3 signaling activation plays a key role in the emergence of cells tolerant to the ALK-TKIs alectinib and brigatinib.

### HER3-mediated resistance with alterations in EMT marker expression is reversible under drug-free conditions

**Fig. 2:**
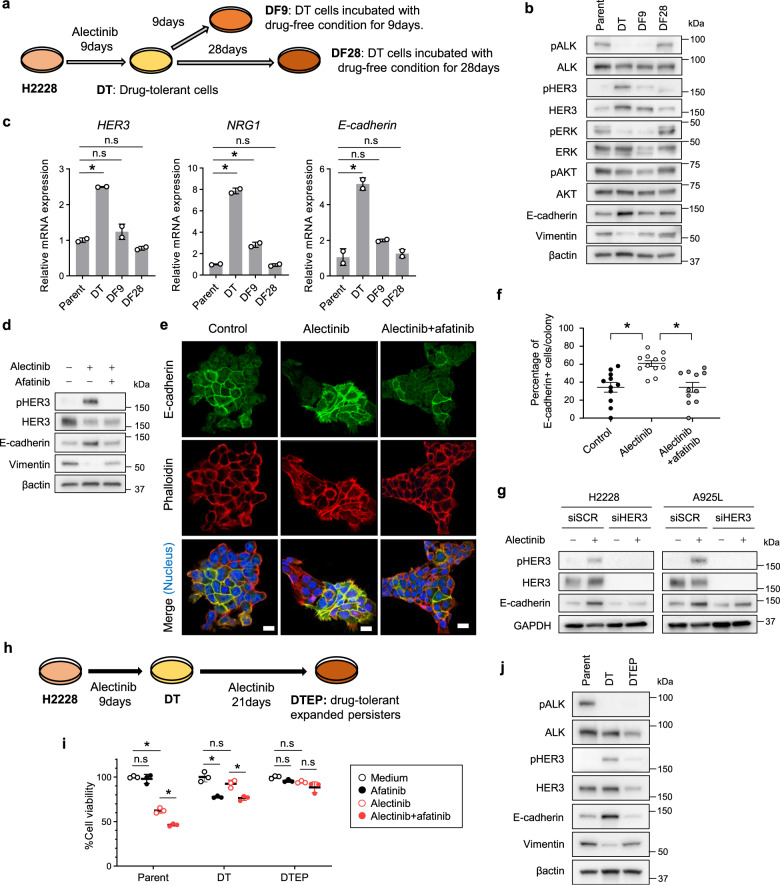
HER3-mediated adaptive resistance with alterations in EMT marker expression is reversible under drug-free conditions. **a** Plated H2228 cells were untreated (left) or treated with alectinib (3 µmol/L) for 9 days (DT cells; left middle) and DT cells were incubated with drug-free-medium for 9 days (DF9; right middle) or 28 days (DF28; right). **b** Western blotting of H2228 cells. **c** qPCR of HER3, NRG1, or E-cadherin in H2228 cells. *P < 0.05 (two-way ANOVA followed by Dunnett’s test). **d** Western blotting of H2228 cells treated with 3 µmol/L alectinib, 100 nmol/L afatinib, or a combination for 72 h. **e** Immunocytochemical analysis of H2228 cells treated with or without alectinib (3 µmol/L) and afatinib (100 nmol/L) for 96 h. **f** Quantification of E-cadherin-positive cells treated with or without alectinib (3 µmol/L) and afatinib (100 nmol/L) for 96 h. *P < 0.05 vs. alectinib-treated cells (Tukey–Kramer method). **g** Western blotting of cells treated with nonspecific control siRNA or *HER3-*specific siRNA and incubated with or without alectinib for 48 h. **h** Plated H2228 cells were untreated (left) or treated with alectinib (3 µmol/L) for 9 days (DT cells; middle), and DT cells were incubated with alectinib (3 µmol/L) for 30 days (drug-tolerant expanded persisters, DTEPs; right). **i** Cell viability MTT assays of H2228 parental, DT, and DTEP cells treated with medium only, 100 nmol/L of alectinib, afatinib, or a combination for 72 h. *P < 0.05 (two-way ANOVA followed by Tukey’s test). **j** Western blotting of cells observed in (**i**). Data are represented as mean ± S.D.

Next, we examined the effect of 9 or 28 days of drug-free (DF) conditions on H2228-DT cells treated with 3 µmol/L alectinib for 9 days (Fig. 2a). Interestingly, DT cells exhibited a different morphology and slower growth compared with that of parental cells, which reverted to the original spindle shape and growth rate following 28 days of DF conditions (Supplementary Fig. 3a, b). Although HER3-mediated DT cell resistance was not easily reversed with respect to alectinib sensitivity and afatinib independence, DT status was eventually reversed following 28 days of DF conditions (Supplementary Fig. 3c). Additionally, DT cells displayed marked total and phosphorylated HER3 expression that was gradually reduced under DF conditions for 9 and 28 days. Notably, DT cells displayed increased E-cadherin and decreased vimentin expression compared with that of parental cells, suggesting that alectinib exposure promotes mesenchymal-to-epithelial transition (MET); however, these expressions levels were reversed under DF conditions for 9 days (Fig. 2b). DT cells also expressed significantly higher E-cadherin, *HER3*, and *NRG1* mRNA levels than those of the parental cells, which was reversed by DF conditions, consistent with western blot results (Fig. 2c).

Furthermore, we revealed that alectinib- or brigatinib-resistant H2228 and A925L DT cells showed high E-cadherin and low vimentin expression compared with that of the parental H2228 and A925L cells (Supplementary Fig. 4a). To evaluate the morphological changes induced by drug exposure, we examined the localization of morphology-related proteins vimentin and E-cadherin following alectinib treatment for 96 h. Immunocytochemistry revealed no overlap between vimentin in the cytoplasm and E-cadherin at the cell membrane, suggesting that E-cadherin-positive cells underwent MET during treatment (Supplementary Fig. 4b). Additionally, western blotting showed that alectinib increased phosphorylated HER3 and E-cadherin expression and decreased vimentin expression in parental H2228 cells, whereas the combination of alectinib and afatinib had only marginal effects on their expression compared with that of controls (Fig. 2d). Consistent with these results, the percentage of E-cadherin-positive cells was significantly higher in alectinib-treated DT H2228 cells than in parental or DT cells treated with a combination of alectinib and afatinib (Fig. 2e, f). Moreover, HER3 knockdown restored the increase in E-cadherin levels in H2228 and A925L parental cells treated with alectinib (Fig. 2g).

We further isolated DT expanded persisters (DTEPs) from H2228 cells exposed to high doses of alectinib (3 µmol/L) for 30 days (Fig. 2h). DTEP cell morphology differed from that of DT cells and reverted to that of the similar parental cells (Supplementary Fig. 4c). Compared with DT cells, DTEP cells were fast-growing and fewer were in the G1 phase of the cell cycle, resulting in a phenotype similar to that of the parental cells (Supplementary Fig. 4d, e). Moreover, DTEP cells were resistant to afatinib alone and in combination with alectinib (Fig. 2i) and displayed decreased total and phosphorylated HER3 expression compared with DT cells (Fig. 2j). Additionally, increased E-cadherin and decreased vimentin expression observed in DT cells was reversed during the transition to DTEP state. We previously showed that EMT via ZEB1 was a mechanism of acquired resistance to ALK-TKIs, and HDAC inhibitor quisinostat could overcome this resistance by reverting EMT^13^. We next evaluated the efficacy of quisinostat in DTEP cells. Western blotting analysis showed that DTEP cells exhibit a decrease of ZEB1 levels, and there was upregulation of E-cadherin upon quisinostat treatment, indicating restored EMT features by quisinostat (Supplementary Fig. 4f, g). Additionally, quisinostat treatment sensitized DTEP cells to alectinib, suggesting that EMT induced resistance to alectinib in the DTEP state.

Taken together, these findings suggest that HER3 activation by ALK inhibition regulates reversible changes in EMT marker expression in ALK-rearranged NSCLC cells.

### ZEB1 regulates MET and HER3 activation in ALK-rearranged NSCLC cells treated with ALK-TKIs

**Fig. 3:**
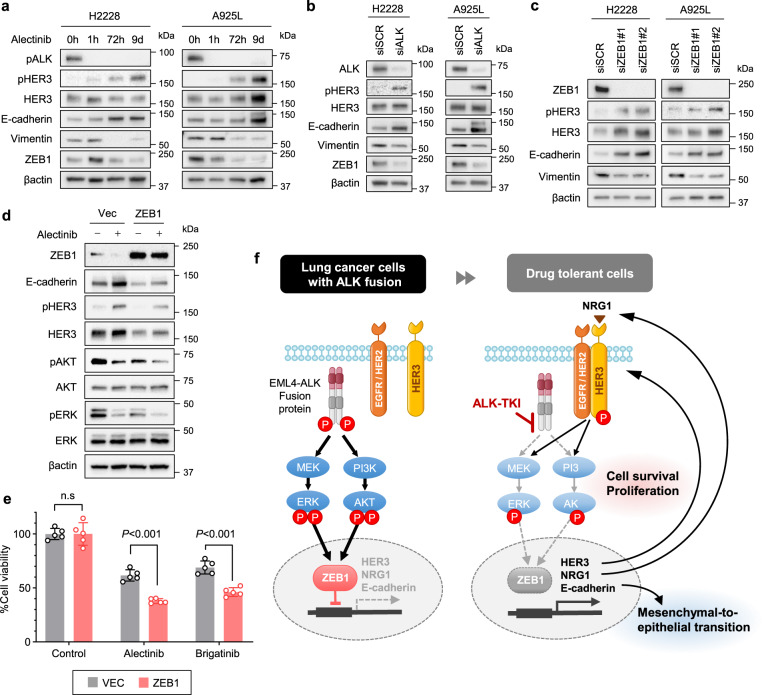
ZEB1 facilitates alterations in EMT marker expression and HER3 activation in ALK-TKI treated cells. **a**–**c** Western blotting of H2228 and A925L cells incubated with or without alectinib (3 µmol/L) for the indicated time (**a**); incubated with nonspecific control siRNA or *ALK-*specific siRNA (**b**); and incubated with nonspecific control siRNA or *ZEB1-*specific siRNAs (#1, #2) (**c**). **d** Western blotting of H2228 cells transfected with a control vector (Vec) or ZEB1-expressing lentiviral plasmids (ZEB1) and treated with alectinib (100 nmol/L) for 48 h; *n* ≥ 2 independent experiments. **e** Cell viability MTT assays of H2228 cells transfected with a control vector (Vec) or ZEB1-expressing lentiviral plasmids (ZEB1) were treated with alectinib (100 nmol/L) for 72 h. Data are represented as mean ± S.D. *P* values were calculated using two-way ANOVA followed by Tukey’s test. **f** Schematic diagram of the drug tolerance mechanisms, including mesenchymal-to-epithelial transition (MET) and HER3 activation via ZEB1, in ALK-rearranged NSCLC cells.

To further understand the link between EMT marker expression and HER3 activation by ALK inhibition, we examined EMT-related protein expression under alectinib exposure. Treatment of H2228 and A925L cells with alectinib for 3 and 9 days decreased expression of the EMT-related transcriptional repressor ZEB1 and vimentin, but exposure for 3 days increased phosphorylated HER3 and E-cadherin levels (Fig. 3a). Consistent with this, specific siRNA-mediated knockdown of *ALK* in H2228 and A925L cells increased E-cadherin and phosphorylated HER3 expression and reduced vimentin and ZEB1 expression (Fig. 3b). Furthermore, the expression of ZEB1 was reduced by specific inhibitors of MAPK/ERK or PI3K/AKT, suggesting that ZEB1 expression is maintained through ALK and its downstream signals, such as MAPK/ERK and PI3K/AKT (Supplementary Fig. 5a).

To assess the effect of ZEB1 on EMT marker expression in NSCLC cells with ALK rearrangements, we investigated the involvement of the ZEB1 feedback loop by regulating its expression. siRNA-mediated knockdown of ZEB1 induced MET-phenotypic changes and HER3 activation in H2228 and A925L cells (Fig. 3c). Following alectinib treatment, ZEB1 overexpression restored the increased expression of phosphorylated HER3 and E-cadherin and enhanced the inhibition of cell viability (Fig. 3d, e). To elucidate the signal transduction pathways induced by HER3 activation, we investigated the effect of downstream molecules such as PI3K/AKT or MEK/ERK on H2228 and A925L cell viability. The MEK inhibitor trametinib or PI3K inhibitor buparlisib yielded additional effects when individually administered with alectinib, indicating that ALK inhibition adversely activates HER3 by modulating MEK/ERK and PI3K/AKT signaling, thus maintaining cell viability (Supplementary Fig. 5b). These results suggest that ZEB1 acts as a key regulator of ALK-TKI tolerance by modulating EMT marker expression and HER3 mediated cell survival signaling in ALK-rearranged NSCLC (Fig. 3f).

### Afatinib sensitizes ALK-rearranged NSCLC cells with a mesenchymal-like phenotype to ALK-TKIs

**Fig. 4:**
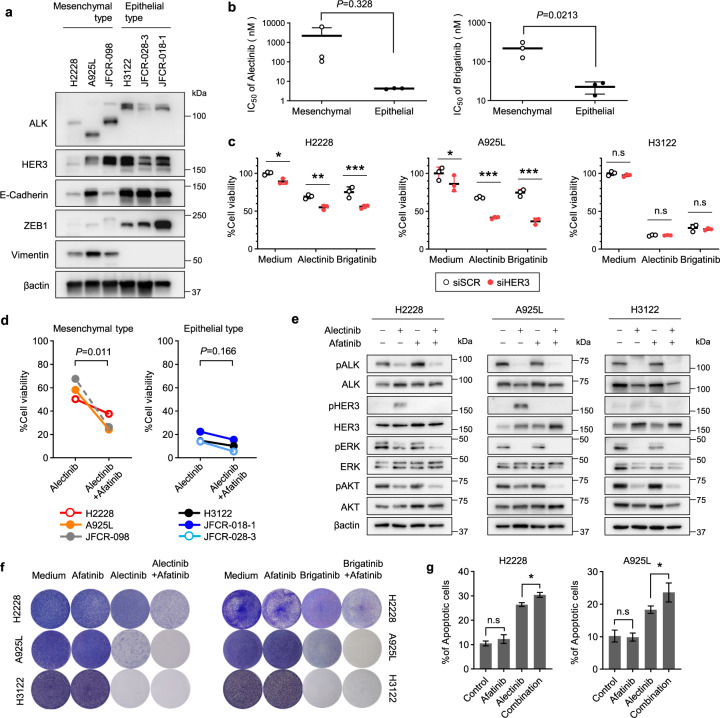
EMT marker expression is correlated with susceptibility to ALK-TKIs. **a** Western blotting of ALK-rearranged NSCLC parental cells. **b** IC_50_ values for alectinib and brigatinib in five ALK-rearranged NSCLC cells. **c** Cell viability MTT assays of H2228 and A925L cells treated with nonspecific control siRNA or HER3-specific siRNAs (#1 and #2) and incubated with or without ALK-TKIs (100 nmol/L) for 72 h. **d** MTT assay of cells treated with alectinib (100 nmol/L) alone or afatinib (100 nmol/L) for 72 h. **e** Western blotting of H2228, A925L, or H3122 parental cells incubated with medium only, alectinib (100 nmol/L), afatinib (100 nmol/L), or a combination for 48 h. **f** Crystal violet staining of cells treated with DMSO, 100 nmol/L of ALK-TKI (alectinib or brigatinib), 100 nmol/L of afatinib, or a combination for 14 days with the drug replenished every 72 h prior to visual examination with crystal violet staining. **g** Apoptotic cell percentages of H2228 and A925L cells, which are annexin V and propidium iodide double-stained, were detected by flow cytometry following treatment with medium, alectinib (100 nmol/L), afatinib (100 nmol/L), or combination for 48 h. Data are represented as mean ± S.D. *P < 0.05, **P < 0.01, ***P < 0.001 [unpaired *t*-tests (**b**, **d**) and two-way ANOVA followed by Tukey’s test (**c, g**)].

We sought to elucidate the correlation between EMT-related protein expression and susceptibility to ALK-TKIs in ALK-rearranged NSCLC cell lines. Although vimentin was expressed in three of the six ALK-rearranged NSCLC cell lines tested, these cell lines were categorized as either having a mesenchymal-like phenotype (low E-cadherin and high vimentin levels) or an epithelial-like phenotype (high E-cadherin and low vimentin levels; Fig. 4a). Importantly, the IC_50_ for both alectinib and brigatinib were significantly higher in cells with mesenchymal features than in those with epithelial features (Fig. 4b). We further investigated the effect of HER3 activation in ALK-rearranged NSCLC cells with different morphological phenotypes by knocking down HER3. Alectinib inhibited the viability of both mesenchymal-like H2228 and A925L cells by 40% and epithelial-like H3122 cells by 80%, whereas HER3 knockdown decreased the viability of H2228 and A925L cells by a further 20–30% compared with that of alectinib treatment for 72 h. In H3122 cells, HER3 knockdown did not show an additional effect on the viability of alectinib-treated cells. Similar observations were made with HER3 knockdown and brigatinib treatment, suggesting that upregulation of the HER3 signaling pathway may promote the survival of a subset of ALK-rearranged NSCLC cells with mesenchymal features when ALK signaling is inhibited by alectinib exposure for 72 h (Fig. 4c).

Next, we evaluated whether afatinib increases the ALK-TKI sensitivity of ALK-rearranged NSCLC cells. Afatinib treatment enhanced the inhibitory effects of alectinib on viability of mesenchymal-like H2228, A925L, and JFCR-098 cells but only exerted marginal effects on the viability of epithelial-like JFCR-018-1, H3122, and JFCR-028-3 cells. Indeed, alectinib (100 nmol/L) had a significantly smaller effect in mesenchymal cell lines treated with afatinib (100 nmol/L) than in those treated with alectinib alone but had marginal effects on the growth of epithelial-like cell lines (Fig. 4d and Supplementary Fig. 6a). The combination index (CI) values of co-treatment with ALK-TKIs and afatinib was less than 1.0, indicating a synergic effect in H2228 and A925L cells (Supplementary Fig. 6b).

To elucidate the mechanism underlying combined therapy with afatinib and ALK-TKIs, we performed western blotting and flow cytometry analysis. Treating H2228 and A925L cells with the ALK-TKIs alectinib or brigatinib for 4 h completely inhibited AKT and ERK phosphorylation, regardless of afatinib treatment, whereas combined treatment with afatinib and alectinib for 48 h remarkably inhibited HER3 and AKT phosphorylation compared with alectinib treatment alone. In contrast, AKT phosphorylation was unaffected in alectinib-treated H3122 cells with or without afatinib for 48 or 4 h (Fig. 4e and Supplementary Fig. 6c). Moreover, the continuous cotreatment of H2228 and A925L, but not H3122 cells, with alectinib or brigatinib and afatinib prevented the emergence of DT cells compared with that of ALK-TKIs alone (Fig. 4f). Flow cytometry analysis showed that the population of apoptotic cells increased with alectinib monotherapy, whereas this population was significantly higher upon treatment in combination with afatinib in H2228 and A925L cells (Fig. 4g).

Together, these results show that cell sensitivity to the ALK-TKIs alectinib or brigatinib is enhanced by cotreatment with afatinib in ALK-rearranged NSCLC cell lines with a mesenchymal-like phenotype, but not in those with an epithelial-like phenotype.

### Cotreatment with afatinib and ALK-TKIs prevents CDX tumor re-growth

**Fig. 5:**
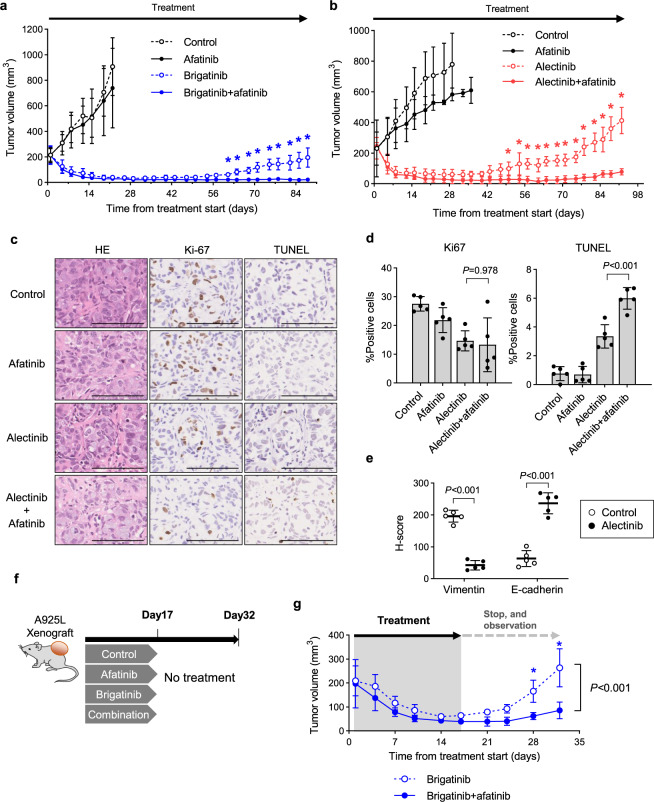
Afatinib with ALK-TKIs prevents CDX tumor regrowth. **a**, **b** A925L cell line-derived xenograft (CDX) tumors (**a**) and H2228 CDX tumors (**b**) were treated with a vehicle (control), ALK-TKI brigatinib (10 mg/kg) or alectinib (6 mg/kg), afatinib (5 mg/kg), or ALK-TKI plus afatinib (5 mg/kg) (*n* = 6) via oral gavage daily. **c** Proliferating and apoptotic cells were quantified by their Ki-67-positive proliferation index (percentage of Ki-67-positive cells) and TUNEL assays, respectively. The scale bar indicates 100 μm. **d** Representative immunohistochemistry images of CDX tumors following staining with specific human Ki-67 and TUNEL antibodies. **e** Quantification of immunostaining-positive cells, as determined by the *H*-score for vimentin and E-cadherin calculated from areas randomly selected from CDX tumors treated with or without alectinib (6 mg/kg) for 4 days. **f** Mice with A925L-CDX tumors were treated with ALK-TKI brigatinib (10 mg/kg) or brigatinib plus afatinib (5 mg/kg) (*n* = 6) by oral gavage daily for 17 days. **g** Tumor regrowth was evaluated on day 32. Tumor volumes were measured from the start of treatment. Data are represented as mean ± S.D. *P < 0.05 [two-way ANOVA followed by Bonferroni’s test (**a**, **b**, **g**) and one-way ANOVA followed by Tukey’s post hoc test (**d, e**)].

We evaluated the effect of afatinib combined with brigatinib is a CDX model using A925L cells. While treatment with afatinib alone only induced a marginal effect on A925L tumor growth, treatment with brigatinib alone caused tumor regression within 1 week, but the tumors regrew within 10 weeks. Similarly, combined treatment with brigatinib and afatinib also caused tumor regression within 1 week; however, the size of the regressed tumors was maintained for 12 weeks (Fig. 5a). No apparent adverse events, including weight loss, were observed during treatment (Supplementary Fig. 7a).

Next, we evaluated the effect of combined afatinib and alectinib treatment in a CDX model using H2228 cells. Treatment with afatinib alone exerted a marginal effect on H2228 tumor growth, whereas alectinib treatment alone caused tumor regression within 1 week, which regrew within 9 weeks, indicating recurrence due to acquired resistance. Co-treatment with alectinib and afatinib also caused tumor regression within 1 week; however, the size of the regressed tumors was maintained for 13 weeks (Fig. 5b). No apparent adverse events, such as weight loss, were observed (Supplementary Fig. 7b).

The number of Ki-67-positive proliferating tumor cells did not differ significantly between the alectinib- or combination-treated tumors derived from H2228 cells; however, there were significantly more TUNEL-positive apoptotic tumor cells in the combination-treated tumors (Fig. 5d). Immunohistochemistry revealed that alectinib-treated tumors exhibited significantly lower *H*-scores for vimentin than non-treated tumors and that *H*-scores for vimentin and ZEB1 were inversely correlated with those of E-cadherin (Fig. 5e and Supplementary Fig. 8a–c). H2228 cell-derived tumors showed homogenous vimentin expression, and while 4-day alectinib treatment led to decreased vimentin expression, regrowing resistant tumors treated with alectinib for 91 days displayed increased heterogenous vimentin expression (Supplementary Fig. 8d). Immunohistochemistry also revealed that tumors treated with alectinib for 91 days displayed significantly higher *H*-scores for vimentin and ZEB1, and lower *H*-scores for E-cadherin than those treated for 4 days (Supplementary Fig. 8e).

To investigate the impact of preventing tolerant cell growth during CDX tumor re-growth, A925L tumor-bearing mice were treated with either brigatinib alone or a combination of brigatinib and afatinib daily via oral gavage until day 17 (Fig. 5f). Although brigatinib treatment decreased tumor size, small tumors were maintained throughout the treatment. Following brigatinib treatment discontinuation, the tumors regrew within 10 days, showing rapid recurrence (Fig. 5g). In contrast, treatment with afatinib plus brigatinib resulted in tumor disappearance. Surprisingly, these tumors showed little regrowth despite discontinuation of the combination treatment for two weeks. No apparent adverse events, including weight loss, were observed during treatment (Supplementary Fig. 7c).

Together, these observations indicate that HER3 activation retains the viability of ALK-TKI-treated cells in vivo and that initial HER3 inhibition improves the response of ALK-rearranged NSCLC tumors to ALK-TKI treatment by promoting apoptosis and preventing recurrence, leading to the potential eradication of tumor activity in ALK-rearranged lung cancer.

### Pre-treatment vimentin expression is associated with poor alectinib outcomes in patients with ALK-rearranged NSCLC

Finally, we retrospectively investigated the correlation between the expression of the mesenchymal-related protein vimentin and the clinical efficacy of alectinib in 34 treatment-naïve patients with ALK-rearranged NSCLC. The probability of cytoplasmic vimentin expression in cells from pre-alectinib-treated tumors was evaluated using immunohistochemical staining (Supplementary Fig. 9a). Out of the 34 pre-treatment ALK-rearranged NSCLC tumor specimens, 18 (53%) lacked vimentin expression and 16 (47%) expressed vimentin (Supplementary Table 1). Following alectinib treatment, patients with vimentin-positive tumors displayed a shorter PFS than those with vimentin-negative tumors (21 and 44 months, respectively; HR = 2.15, 95% confidence interval (CI) = 0.75–6.35*, P* = 0.126; Supplementary Fig. 9b) and displayed a significantly shorter overall survival (OS) following alectinib treatment (median OS = 54 months vs. not reached (N.R.); HR = 8.70, 95% CI = 1.49–50.86, *P* = 0.016; Supplementary Fig. 9c). Together, these findings suggest that high pre-treatment vimentin expression may correlate with a poor response to alectinib and poor survival in patients with ALK-rearranged NSCLC.

## Discussion

Although oncogene-driven cancer cells display good initial responses to molecular targeted therapy, a small percentage of cells can survive and expand, leading to acquired drug resistance and tumor heterogeneity, ultimately enhancing tumor recurrence^19,20^. A recent study showed that heterogeneous pretreatment tumor evolution was closely associated with rapid alectinib resistance in ALK-rearranged NSCLC^21^. Several bypass pathway activations (MAP2K1, SRC, EGFR, and PI3K) have been reported to promote ALK-TKI resistance^12,22,23^; however, current targeted approaches have failed to conquer acquired ALK-TKI resistance. Previously, we detected ALK-mutant L1196M and EMT in a single crizotinib-resistant lesion from a patient with ALK-rearranged NSCLC^13^, indicating that these are concomitant mechanisms that can independently induce crizotinib resistance in patients with ALK-rearranged NSCLC. As it can be difficult to tackle drug resistance in ALK-rearranged NSCLC tumors following ALK-TKI interventions due to tumor heterogeneity, it may be best to first limit the number of DT cells based on knowledge of the molecular mechanisms underlying intrinsic resistance and early ALK-TKI refractoriness^4^. Herein, we examined the major feedback mechanisms in NSCLC cells exposed to ALK-TKIs to identify an adaptive response to HER3 activation, which binds to the HER family proteins, EGFR and HER2. Aberrant HER3 expression is involved in carcinogenesis and the progression of diverse human cancers^24^, and its activation has been observed in the active PI3K/AKT signaling feedback loop in HER2-driven breast cancer cells treated with EGFR or HER2 inhibitors^25^. Previously, we showed that HER3 binds to anexelekto (AXL), maintains cell survival, and induces cell tolerance to the EGFR-TKI osimertinib via the ERK-SPRY4 axis in EGFR mutant-NSCLC cells^17^. Moreover, HER3 re-activation with or without ligand stimulation was found to induce resistance to several ALK-TKIs in ALK-rearranged NSCLC^26,27^. To our knowledge, this study is the first to report that HER3 activation promotes tolerance to molecularly targeted agents and maintains cell survival in NSCLC with driver gene alterations. The pan-HER inhibitor afatinib was approved as a first-line clinical treatment for patients with advanced EGFR mutant NSCLC^28^ and displays good tolerance and safety. We found that afatinib potently suppressed the emergence and maintenance of ALK-rearranged NSCLC cells tolerant to alectinib or brigatinib, thus afatinib shows promise for drug repositioning to treat ALK-rearranged NSCLC patients and should be evaluated in future clinical studies. Although we revealed the mechanisms underlying HER3 activation and escape from ALK-TKI tolerance in ALK-rearranged NSCLC cells, this study had some limitations. Particularly, the precise mechanistic details, including specific ALK binding partners, their direct downstream molecules related to ALK-TKI tolerance, and the best duration for HER3 inhibition intervention, remain unclear. Thus, further experiments are warranted to clarify this mechanism and are underway in our laboratory.

Alterations in EMT marker expression play critical roles in developmental processes, such as facilitating cancer invasion, metastasis, and drug resistance. Although EMT can induce resistance to targeted therapies, including ALK-TKIs, its reversal restores drug sensitivity. Conversely, MET is essential for the colonization and metastasis of differentiated carcinomas^29^ and has been the focus of an increasing number of studies. MET is induced by the inhibition of SHP2, BMP2, β-catenin, and recombinant human BMP7^30,31^, while AKT activation is negatively related to MET in breast carcinoma^31^. Herein, alectinib- or brigatinib-DT cells from mesenchymal-like ALK-rearranged NSCLC exhibited MET with increased E-cadherin expression and reduced mesenchymal marker (ZEB1 and vimentin) expression, but reverted to a mesenchymal phenotype following HER3 inhibition or ZEB1 overexpression. E-cadherin re-expression may play a role in MET induction^32^ and its transcriptional repressor ZEB1 is known to play key roles in EMT and drug resistance in several tumor cell lines^33,34^. Previously, we showed that EMT could be induced by reducing the expression of miR-200 family members, such as miR-200c and miR-141, thus increasing ZEB1 and decreasing E-cadherin expression in ALK-TKI-resistant ALK-rearranged NSCLC cells^13^. Moreover, ZEB1 represses HER3 promoter activity by suppressing NOTCH1 in EGFR-mutated lung cancer cells^35^. Our findings suggest that feedback loops for HER3 reactivation via ZEB1 may contribute toward tumor differentiation in DT and reversible ALK-rearranged NSCLC cells and reveal a novel DT system with MET related to HER3 activation in cancer. Interestingly, ALK-rearranged NSCLC tumors exhibit a high EMT phenotype frequency with decreased E-cadherin and increased vimentin expression compared to NSCLC tumors with other genotypes^36^, whereas ALK activation is known to retain a mesenchymal phenotype by downregulating E-cadherin^37^. Our observations in clinical specimens demonstrated that vimentin expression is related to poor outcomes in naïve ALK-rearranged NSCLC patients, indicating that a novel therapeutic strategy is required for these patients. Recent studies showed that a DT state is an alternative route to acquiring resistance^16,38,39^. Herein, we showed that DTEP cells reversed morphological changes and promoted drug resistance independent of HER3 activation, consistent with mouse xenograft models, suggesting that adaptive resistance via HER3 and MET may not directly lead to acquired resistance to ALK-TKIs. These novel observations shed light on the dynamic morphological changes from adaptative resistance toward acquired resistance to molecular-targeted agents and supported the notion that refractory tumors pose a challenge to being completely cured via the emergence of drug tolerant clones. Moreover, our findings suggested that HER3-targeted therapy to conquer DT mechanisms at the initial phase may be promising to eradicate the emergence of tumor heterogeneity. However, it remains unclear how adaptive resistance to ALK inhibitors via HER3 activation and MET is related to the selection of epigenetically predefined subclones or true tumor cell plasticity. Further investigations are required to elucidate the underlying mechanisms.

In conclusion, we revealed that HER3 reactivation was biologically significant in ALK-rearranged NSCLC cells treated with the ALK-TKIs alectinib and brigatinib. Moreover, we demonstrated that HER3 plays a pivotal role in intrinsic ALK-TKI resistance in ALK-rearranged NSCLC and the emergence of DT cells. Additionally, ALK-TKI tolerance induced MET, which was reversed by HER3 inhibition. Together, these results suggest that initial treatment with a combination of ALK-TKIs and afatinib may prevent the development of intrinsic ALK-TKI resistance and the emergence of DT cells with MET in ALK-rearranged NSCLC, leading to the potential eradication of these tumors.

## Methods

### Cell culture and reagents

H2228 (EML4–ALK variant 3a/b E6; A20) cells were purchased from the American Type Culture Collection (Manassas, VA, USA). A925L (EML4–ALK variant 5a, E2: A20) was established from a surgical specimen of the EML4–ALK-positive NSCLC patient and kindly provided by Fumihiro Tanaka of the Second Department of Surgery, University of Occupational and Environmental Health, Japan^40^. The H3122 human lung adenocarcinoma cell line, with EML4–ALK fusion protein variant1 (E13; A20), was kindly provided by Dr. Jeffrey A. Engelman of the Massachusetts General Hospital Cancer Center (Boston, MA)^6^. These cell lines were maintained in Roswell Park Memorial Institute (RPMI) 1640 medium with 10 % fetal bovine serum (FBS), penicillin (100 U/mL), and streptomycin (100 µg/mL) in a humidified 5% CO_2_ incubator at 37 °C. The EML4–ALK-positive NSCLC patient-derived cell lines JFCR-018-1 and JFCR-028-3 were cultured in a medium containing equal proportions of RPMI1640 and Ham’s F12 (Wako) and supplemented with 15% FBS and 1 × antibiotic–antimycotic mixed stock solution (Wako). The EML4–ALK-positive NSCLC patient-derived cell lines JFCR-098 was cultured in StemPro-hESC + Y medium [1:1 (v/v) Ham’s F12-DMEM + GlutaMAX, supplemented with 1× StemPro, 1.6% bovine serum albumin, 8 ng/ml bFGF, 100 μM 2-Mercapto-ethanol, 10 μM Y-27632] supplemented with 1× antibiotic–antimycotic mixed stock solution. All cells were passaged for less than three months before being renewed with frozen early-passage stocks and were regularly screened for mycoplasma using a MycoAlert Mycoplasma Detection Kit (Lonza, Basel, Switzerland). Alectinib, brigatinib, crizotinib, lorlatinib, afatinib, trametinib, linsitinib, and quisinostat were obtained from Selleck Chemicals (Houston, TX).

### Cell viability assay

Tumor cells (2–3 × 10^3^ cells/100 μL/well) were cultured with the indicated compound for 72 h, after which cell viability was determined using MTT assays (Sigma-Aldrich, St. Louis, MO) according to the manufacturer’s instructions. Absorbance was measured using a microplate reader at the test and reference wavelengths of 570 and 630 nm, respectively. Percentage growth was determined relative to untreated controls. Cells were also treated at 37 °C with the indicated agents for two weeks, with the drugs replenished every 72 h.

### Human phospho-receptor tyrosine kinases (RTK) array

The relative phosphorylation levels of 49 RTKs and two related proteins were measured using a Human Phospho-RTK Array Kit (R&D Systems, Minneapolis, MN, USA) according to the manufacturer’s instructions, with slight modifications. Briefly, cells were cultured in RPMI 1640 containing 10% FBS and lysed in array buffer prior to reaching confluence. The arrays were blocked with blocking buffer and incubated overnight with 300 μg of cell lysate at 4 °C before being washed, incubated with horseradish peroxidase (HRP)-conjugated phospho-kinase antibodies, and treated with SuperSignal West Dura Extended Duration Substrate (Pierce Biotechnology, Rockford, IL, USA).

### Western blotting

Proteins (25 μg aliquots) were resolved by SDS polyacrylamide gel electrophoresis (Bio-Rad, Hercules, CA, USA) and total protein (1000 μg) was immunoprecipitated using the indicated antibodies. The procedure for western blotting was as previously described^17^. All blots derive from the same experiment and were processed in parallel.

### Antibodies used in this study

The following primary antibodies were used for indicated experiments; western blotting: p-ALK (Tyr1604), t-ALK, p-HER3, t-HER3, β-actin (13E5), t-HER2, p-Akt, t-Akt, E-cadherin, Vimentin, TCF8/ZEB1, and GAPDH, (1:1000 dilution; Cell Signaling Technology), and p-Erk1/2 (Thr202/Tyr204), t-ERK1/2, and t-EGFR (1:1000 dilution; R&D systems); immunohistochemistry: vimentin (ACR 048A, C; Biocare Medical, Concord, CA, USA), E-cadherin (M3612; Dako, Santa Clara, CA, USA) and ZEB1 (ab180905; Abcam, Cambridge, UK).

### Microarray gene expression study

Total RNA was extracted using NucleoSpin^®^ RNA Plus (Takara Bio, Shiga, Japan) according to the manufacturer’s instructions. cDNA was synthesized using GeneChip® WT PLUS Reagent Kit (Thermo Fisher Scientific, Waltham, MA) and hybridized to the Clariom S Human assay (Affymetrix, Santa Clara, CA). The arrays were scanned and normalized with the Signal Space Transformation-Robust Multi-array Average algorithm (SST-RMA) using the Affymetrix GeneChip Command Console (Thermo Fisher Scientific). Differential gene expression analysis was performed using Transcriptome Analysis Console Software 4.0.2.15 (Thermo Fisher Scientific). Functional enrichment analysis of the differentially expressed genes and KEGG pathway analysis^41^ were performed using gene set enrichment analysis (GSEA; http://software.broadinstitute.org/gsea/msigdb/)^42^. “KEGG_ERBB_SIGNALING_PATHWAY” was used for gene set annotation. Log_2_-transformed expression data were applied to GSEA and analyzed using Dif_of_Classes as a metric and 1,000 permutations of specific gene sets.

### Real-time PCR analysis

Total RNA was extracted using NucleoSpin^®^ RNA Plus (Takara Bio, Shiga, Japan) and cDNA was synthesized using PrimeScript™ RT Master Mix (Perfect Real Time; Takara Bio) according to the respective manufacturer’s instructions. Real-time PCR was performed using a TaKaRa PCR Thermal Cycler Dice^®^ (Takara Bio) and SYBR Fast qPCR kit (Kapa Biosystems, Cape Town, South Africa) using the following amplification protocol: initial incubation at 95 °C for 10 min, 40 cycles at 95 °C for 15 s and 60 °C for 1 min, followed by melting curve analysis. Gene expression was calculated from relative standard curves, normalized to *GAPDH*, and analyzed using the 2^−ΔΔCT^ method^43^. The following primer sequences were used: *HER3* (F: 5′-TGC TGA GAA CCA ATA CCA GAC A-3′, R: 5′-CTG TCA CTT CAC GAA TCC ACT G-3′); *NRG1* (F: 5′-AGT CCT TCG GTG TGA AAC CAG-3′, R: 5′-TGC GAA GTT CTG ACT TCC CTG-3′); *E-cadherin* (F: 5′-GAA CGC ATT GCC ACA TAC AC-3′, R: 5′-GAA TTC GGG CTT GTT GTC AT-3′); and *GAPDH* (F: 5′-CTG CAC CAA CTG CTT AG-3′, R: 5′-TGA AGT CAG AGG AGA CCA CC-3′).

### Immunocytochemistry

H2228 cells were cultured at 37 °C in 5% CO_2_ on fibronectin-coated chamber slides before being stimulated with alectinib (3 µmol/L) with or without afatinib (0.1 µmol/L) for 96 h. Cells were fixed with 4% paraformaldehyde for 15 min at 25 ± 1 °C, permeabilized with 0.02% Triton in PBS, and incubated with primary antibodies against E-cadherin (#3195; Cell Signaling Technology) and vimentin (#ab92547; Abcam), followed by FITC- or Alexa Fluor 568-conjugated secondary antibodies (Dako, Santa Clara, CA). As the same antibodies were used to identify vimentin and E-cadherin, we used a Zenon Alexa Fluor 488 rabbit IgG-labeling kit (Z25002: Thermo Fisher Scientific) to avoid cross-reactivity between secondary antibodies. To visualize actin fibers, cells were treated with TRITC-conjugated phalloidin (Sigma-Aldrich), nuclei were counterstained with 4′,6-diamidino-2-phenylindole (DAPI) or DRAQ5 (DR50050; BioStatus, Leicestershire, UK; 1:2000), and mounted using Prolong-Gold (Thermo Fisher Scientific). Images were obtained via confocal microscopy (FV1000; Olympus, Tokyo, Japan). E-cadherin-positive cells were quantified as the number of cells with positive staining over their entire surface and calculated as a percentage of the total cells per colony (*n* = ≥15 colonies per group).

### siRNA transfection

Duplexed Silencer^®^ Select siRNAs for HER3 (#1) and ZEB1 (#1, #2) and Stealth RNAi for HER3 (#2) were purchased from Invitrogen (Carlsbad, CA, USA). ALK-targeted siRNAs were purchased from Santa-Cruz Biotechnology (Dallas, TX, USA). Cells were transfected with these siRNAs using Lipofectamine RNAi-MAX (Invitrogen) according to the manufacturer’s instructions. In all experiments, Silencer® Select siRNA for negative control #1 (Invitrogen) was used as the scrambled control. HER3, ZEB1, and ALK knockdown were confirmed via western blot analysis.

### Plasmid construction

pWPXL plasmid-expressing empty vectors (vector) and human ZEB1 (ZEB1) were　purchased from Addgene (Watertown, MA, USA) and transfected into A925L cells using Lipofectamine 3000 (Invitrogen) transfection reagent according to the manufacturer’s instructions. The cells were then selected using G418 sulfate (Nacali Tesque, Inc., Kyoto, Japan).

### Apoptotic cell death analysis

H2228 and A925L cells (1 × 10^5^) were treated with or without alectinib (100 nmol/L) and/or afatinib (100 nmol/L) for 48 h. All floating and adherent cells were collected and incubated with Annexin V-FITC and propidium iodide (PI) for 15 min at 25 °C in the dark. Then, samples were analyzed using BD Accuri C6 Plus Flow Cytometer (Becton, Dickinson & Company, Franklin Lakes, NJ, USA) and data were analyzed with FlowJo^®^ software (FlowJo LLC, Ashland, OR, USA), as previously reported^44^.

### Cell-line-derived xenograft (CDX) models

H2228 and A925L cell suspensions in PBS (5 × 10^6^ cells) were injected subcutaneously into the flanks of 5-week-old male C.B-17/Icr-scid/scidJcl mice with severe combined immunodeficiency (Clea Japan, Tokyo, Japan). Once the mean tumor volume had reached approximately 100–200 mm^3^, mice have administrated the targeted drugs via daily oral gavage. Bodyweight was measured twice weekly and general condition was monitored daily. Tumors were measured twice weekly using calipers and their volume calculated as (width^2^ × length)/2. Mouse experimental protocols were approved by the institutional review board of Kyoto Prefectural University of Medicine (Kyoto, Japan; approval no. M29–529). According to institutional guidelines, animal surgery was performed following anesthesia with sodium pentobarbital, and efforts were made to minimize animal suffering.

### Tumor histological analyses

Formalin-fixed, paraffin-embedded tissue sections (4 μm thick) were deparaffinized and antigens were retrieved by microwaving the tissue sections in 10 mM citrate buffer (pH 6.0). Proliferating cells were detected by incubation at 37 °C in 5% CO_2_ with Ki-67 antibodies (Clone MIB-1; GA62661-2, Dako), as described previously^17^. Apoptosis was quantitated using the terminal deoxynucleotidyl transferase-mediated dUTP-biotin nick-end labeling (TUNEL) method, according to the manufacturer’s instructions. Based on their expression patterns, tumor cells from the tissue specimens were separately evaluated for vimentin, E-cadherin, and ZEB1 expression. After incubation with primary and secondary antibodies under the manufacturers’ recommended conditions and treatment with a Vectastain ABC Kit (Vector Laboratories, Burlingame, CA, USA), peroxidase activity was visualized using 3,3′-diaminobenzidine (DAB) as a chromogen and the sections were counterstained with hematoxylin. Immunohistochemical studies have shown that vimentin is present primarily in the cytoplasm, E-cadherin is mainly present in the membrane and cytoplasm, and ZEB1 is present in the nucleus with varying staining intensity. Therefore, we quantified the expression of vimentin, ZEB1, and E-cadherin as either negative (0), weak (1+), moderate (2+), or strong (3+) in comparison with vascular endothelial cells as an internal control and evaluated cells with each intensity in five randomly selected fields (0.1 mm^2^/section) in specimens from the CDX models.

### Quantification of immunohistochemistry results

The five areas containing the most positively stained cells in each section were selected for histological quantitation using light microscopy at 400 × magnification, as described previously^45^. The intensity and percentage of positive cells were determined using the *H*-score method^46^ as follows: Positive cells = 1× (% of 1+ cells) + 2× (% of 2+ cells) + 3× (% of 3+ cells).

### Patients

ALK-rearranged tumor specimens were obtained from 34 patients with NSCLC at University Hospital, Kyoto Prefectural University of Medicine (Kyoto, Japan), Japanese Red Cross Kyoto Daiichi Hospital (Kyoto, Japan), Japanese Red Cross Kyoto Daini Hospital (Kyoto, Japan), and Niigata University Hospital (Niigata, Japan) prior to alectinib treatment. To quantify vimentin expression in tumor tissue obtained from ALK-rearranged NSCLC patients, we classified specimens with whole-cell vimentin expression as either “vimentin-negative” or “vimentin-positive” and observed the staining rates in positive tumor cells. The study was conducted in accordance with the Declaration of Helsinki. All patients provided written informed consent and each hospital study was approved by the institutional review board of University Hospital, Kyoto Prefectural University of Medicine (approval No. ERB-C-1603).

### Statistical analysis

Data from the MTT assays and xenograft tumor progression were expressed as the mean ± SD and the mean ± standard error, respectively. Significant differences were analyzed using two-way ANOVA, unpaired *t* tests, one-way ANOVA, Pearson’s correlation coefficients, and linear regression analysis using Prism 8.0 (GraphPad Software, San Diego, CA, USA), with two-sided *P*-values of <0.05 considered significant.

### Reporting summary

Further information on research design is available in the Nature Research Reporting Summary linked to this article.

## Supplementary information

REPORTING SUMMARY

Sup-Figures

## Data Availability

Transcriptomic data obtained from the microarray analysis of parental cells and DT cells derived from H2228 and A925L have been deposited in Gene Expression Omnibus (GEO) under the accession code GSE188406. The histology images and patient survival data are not publicly available due to containing information that could compromise research participant privacy. Qualified researchers can apply for access to these data by signing a data usage agreement. The other data that support the findings of this study are available on request from the corresponding author.

## References

[1] Miller KD (2018). Cancer statistics for hispanics/latinos, 2018. CA Cancer J. Clin..

[2] Govindan R (2006). Changing epidemiology of small-cell lung cancer in the United States over the last 30 years: analysis of the surveillance, epidemiologic, and end results database. J. Clin. Oncol..

[3] Li T, Kung HJ, Mack PC, Gandara DR (2013). Genotyping and genomic profiling of non-small-cell lung cancer: implications for current and future therapies. J. Clin. Oncol..

[4] Recondo G, Facchinetti F, Olaussen KA, Besse B, Friboulet L (2018). Making the first move in EGFR-driven or ALK-driven NSCLC: first-generation or next-generation TKI?. Nat. Rev. Clin. Oncol..

[5] Soda M (2007). Identification of the transforming EML4-ALK fusion gene in non-small-cell lung cancer. Nature.

[6] Koivunen JP (2008). EML4-ALK fusion gene and efficacy of an ALK kinase inhibitor in lung cancer. Clin. Cancer Res..

[7] Shaw AT (2013). Crizotinib versus chemotherapy in advanced ALK-positive lung cancer. N. Engl. J. Med..

[8] Hida T (2017). Alectinib versus crizotinib in patients with ALK-positive non-small-cell lung cancer (J-ALEX): an open-label, randomised phase 3 trial. Lancet.

[9] Peters S (2017). Alectinib versus crizotinib in untreated ALK-positive non-small-cell lung cancer. N. Engl. J. Med..

[10] Camidge DR (2018). Brigatinib versus crizotinib in ALK-positive non-small-cell lung cancer. N. Engl. J. Med..

[11] Solomon BJ (2018). Lorlatinib in patients with ALK-positive non-small-cell lung cancer: results from a global phase 2 study. Lancet Oncol..

[12] Katayama R (2018). Drug resistance in anaplastic lymphoma kinase-rearranged lung cancer. Cancer Sci..

[13] Fukuda K (2019). Epithelial-to-mesenchymal transition is a mechanism of ALK inhibitor resistance in lung cancer independent of ALK mutation status. Cancer Res..

[14] Kim DW (2017). Brigatinib in patients with crizotinib-refractory anaplastic lymphoma kinase-positive non-small-cell lung cancer: a randomized, multicenter Phase II trial. J. Clin. Oncol..

[15] Shaw AT (2019). ALK resistance mutations and efficacy of lorlatinib in advanced anaplastic lymphoma kinase-positive non-small-cell lung cancer. J. Clin. Oncol..

[16] Sharma SV (2010). A chromatin-mediated reversible drug-tolerant state in cancer cell subpopulations. Cell.

[17] Taniguchi H (2019). AXL confers intrinsic resistance to osimertinib and advances the emergence of tolerant cells. Nat. Commun..

[18] Arasada RR (2018). Notch3-dependent β-catenin signaling mediates EGFR TKI drug persistence in EGFR mutant NSCLC. Nat. Commun..

[19] Suda K (2016). Heterogeneity in resistance mechanisms causes shorter duration of epidermal growth factor receptor kinase inhibitor treatment in lung cancer. Lung Cancer.

[20] Jamal-Hanjani M (2017). Tracking the evolution of non-small-cell lung cancer. N. Engl. J. Med..

[21] Makimoto G (2019). Rapid acquisition of alectinib resistance in ALK-positive lung cancer with high tumor mutation burden. J. Thorac. Oncol..

[22] Katayama R (2012). Mechanisms of acquired crizotinib resistance in ALK-rearranged lung cancers. Sci. Transl. Med..

[23] Gainor JF (2016). Molecular mechanisms of resistance to first- and second-generation ALK inhibitors in ALK-rearranged lung cancer. Cancer Discov..

[24] Gala K, Chandarlapaty S (2014). Molecular pathways: HER3 targeted therapy. Clin. Cancer Res..

[25] Sergina NV (2007). Escape from HER-family tyrosine kinase inhibitor therapy by the kinase-inactive HER3. Nature.

[26] Tanizaki J (2012). Activation of HER family signaling as a mechanism of acquired resistance to ALK inhibitors in EML4-ALK-positive non-small cell lung cancer. Clin. Cancer Res..

[27] Dong X, Fernandez-Salas E, Li E, Wang S (2016). Elucidation of resistance mechanisms to second-generation ALK inhibitors alectinib and ceritinib in non-small cell lung cancer cells. Neoplasia.

[28] Yang JC (2015). Afatinib versus cisplatin-based chemotherapy for EGFR mutation-positive lung adenocarcinoma (LUX-Lung 3 and LUX-Lung 6): analysis of overall survival data from two randomised, phase 3 trials. Lancet Oncol..

[29] Brabletz T (2012). EMT and MET in metastasis: where are the cancer stem cells?. Cancer Cell.

[30] Yao D, Dai C, Peng S (2011). Mechanism of the mesenchymal-epithelial transition and its relationship with metastatic tumor formation. Mol. Cancer Res..

[31] Zhou XD, Agazie YM (2008). Inhibition of SHP2 leads to mesenchymal to epithelial transition in breast cancer cells. Cell Death Differ..

[32] Wells A, Yates C, Shepard CR (2008). E-cadherin as an indicator of mesenchymal to epithelial reverting transitions during the metastatic seeding of disseminated carcinomas. Clin. Exp. Metastasis.

[33] Thiery JP, Acloque H, Huang RY, Nieto MA (2009). Epithelial-mesenchymal transitions in development and disease. Cell.

[34] Kalluri R, Weinberg RA (2009). The basics of epithelial-mesenchymal transition. J. Clin. Invest..

[35] Zhang T (2016). A genetic cell context-dependent role for ZEB1 in lung cancer. Nat. Commun..

[36] Kim H (2013). A comprehensive comparative analysis of the histomorphological features of ALK-rearranged lung adenocarcinoma based on driver oncogene mutations: frequent expression of epithelial-mesenchymal transition markers than other genotype. PLoS ONE.

[37] Voena C (2016). Oncogenic ALK regulates EMT in non-small cell lung carcinoma through repression of the epithelial splicing regulatory protein 1. Oncotarget.

[38] Shah KN (2019). Aurora kinase A drives the evolution of resistance to third-generation EGFR inhibitors in lung cancer. Nat. Med..

[39] Ramirez M (2016). Diverse drug-resistance mechanisms can emerge from drug-tolerant cancer persister cells. Nat. Commun..

[40] Sugaya M (2002). Establishment of 15 cancer cell lines from patients with lung cancer and the potential tools for immunotherapy. Chest.

[41] Kanehisa M, Goto S (2000). KEGG: Kyoto encyclopedia of genes and genomes. Nucleic Acids Res..

[42] Subramanian A (2005). Gene set enrichment analysis: a knowledge-based approach for interpreting genome-wide expression profiles. Proc. Natl Acad. Sci. USA.

[43] Livak KJ, Schmittgen TD (2001). Analysis of relative gene expression data using real-time quantitative PCR and the 2(-Delta Delta C(T)) Method. Methods.

[44] Yang S (2016). A HSP60-targeting peptide for cell apoptosis imaging. Oncogenesis.

[45] Okura N (2020). ONO-7475, a novel AXL inhibitor, suppresses the adaptive resistance to initial EGFR-TKI treatment in EGFR-mutated non-small lung cancer. Clin. Cancer Res..

[46] Yano S (2011). Hepatocyte growth factor expression in EGFR mutant lung cancer with intrinsic and acquired resistance to tyrosine kinase inhibitors in a Japanese cohort. J. Thorac. Oncol..

