# Supplementary material for: HER3 activation contributes toward the emergence of ALK inhibitor-tolerant cells in ALK-rearranged lung cancer with mesenchymal features
Source: NPJ Precis Oncol. 2022 Jan 18;6:5. doi: 10.1038/s41698-021-00250-8 (PMC8766605; doi:10.1038/s41698-021-00250-8)
Supplement: Supplementary file 1 — REPORTING SUMMARY [file 41698_2021_250_MOESM1_ESM.pdf]

nature research

Corresponding author(s): Tadaaki Yamada

Last updated by author(s): Sep-21-2021

Reporting Summary

Nature Research wishes to improve the reproducibility of the work that we publish. This form provides structure for consistency and transparency in reporting. For further information on Nature Research policies, see Authors & Referees and the Editorial Policy Checklist.

Statistics

For all statistical analyses, confirm that the following items are present in the figure legend, table legend, main text, or Methods section.

n/a	Confirmed	
☐	☒	The exact sample size (*n*) for each experimental group/condition, given as a discrete number and unit of measurement
☐	☒	A statement on whether measurements were taken from distinct samples or whether the same sample was measured repeatedly
☐	☒	The statistical test(s) used AND whether they are one- or two-sided<br>*Only common tests should be described solely by name; describe more complex techniques in the Methods section.*
☐	☒	A description of all covariates tested
☐	☒	A description of any assumptions or corrections, such as tests of normality and adjustment for multiple comparisons
☐	☒	A full description of the statistical parameters including central tendency (e.g. means) or other basic estimates (e.g. regression coefficient) AND variation (e.g. standard deviation) or associated estimates of uncertainty (e.g. confidence intervals)
☐	☒	For null hypothesis testing, the test statistic (e.g. *F*, *t*, *r*) with confidence intervals, effect sizes, degrees of freedom and *P* value noted<br>*Give P values as exact values whenever suitable.*
☒	☐	For Bayesian analysis, information on the choice of priors and Markov chain Monte Carlo settings
☒	☐	For hierarchical and complex designs, identification of the appropriate level for tests and full reporting of outcomes
☒	☐	Estimates of effect sizes (e.g. Cohen's *d*, Pearson's *r*), indicating how they were calculated

Our web collection on statistics for biologists contains articles on many of the points above.

Software and code

Policy information about availability of computer code

Data collection	no software was used.
Data analysis	GraphPad Prism Ver. 8.0 (GraphPad Software, Inc., San Diego, CA, USA)

For manuscripts utilizing custom algorithms or software that are central to the research but not yet described in published literature, software must be made available to editors/reviewers. We strongly encourage code deposition in a community repository (e.g. GitHub). See the Nature Research guidelines for submitting code & software for further information.

Data

Policy information about availability of data

All manuscripts must include a data availability statement. This statement should provide the following information, where applicable:

- Accession codes, unique identifiers, or web links for publicly available datasets
- A list of figures that have associated raw data
- A description of any restrictions on data availability

The authors declare that all data supporting the findings of this study are available within the paper and its supplementary information files.

Field-specific reporting

Please select the one below that is the best fit for your research. If you are not sure, read the appropriate sections before making your selection.

☒ Life sciences ☐ Behavioural & social sciences ☐ Ecological, evolutionary & environmental sciences

For a reference copy of the document with all sections, see nature.com/documents/nr-reporting-summary-flat.pdf

Life sciences study design

All studies must disclose on these points even when the disclosure is negative.

Sample size	This research mainly targets for experimental research.
Data exclusions	Exclusion criteria is none.
Replication	Each experiment was independently performed at least twice.
Randomization	In mice experiments, the mice were transferred to the animal facility at Kyoto Prefectural University of Medicine and randomized once their mean tumor volume reached the each indicated volume.
Blinding	Our mice experiments could not blind to each mice groups.

Reporting for specific materials, systems and methods

We require information from authors about some types of materials, experimental systems and methods used in many studies. Here, indicate whether each material, system or method listed is relevant to your study. If you are not sure if a list item applies to your research, read the appropriate section before selecting a response.

Materials & experimental systems

n/a	Involved in the study	
☐	☒	Antibodies
☐	☒	Eukaryotic cell lines
☒	☐	Palaeontology
☐	☒	Animals and other organisms
☐	☒	Human research participants
☒	☐	Clinical data

Methods

n/a	Involved in the study	
☒	☐	ChIP-seq
☐	☒	Flow cytometry
☒	☐	MRI-based neuroimaging

Antibodies

Antibodies used

p-ALK, t-ALK, p-HER3, t-HER3, β-actin, t-HER2, p-Akt, t-Akt, E-cadherin, Vimentin, TCF8/ZEB1, GAPDH, (Cell Signaling Technology)
p-Erk1/2, t-ERK1/2, t-EGFR (1:1000 dilution; R&D systems)
vimentin (ACR 048 A, C; Biocare Medical, Concord, CA, USA)
E-cadherin (M3612; Dako, Santa Clara, CA, USA)
ZEB1 (ab180905; Abcam, Cambridge, UK).

Validation

p-ALK (Tyr1604), t-ALK, p-HER3, t-HER3, β-actin (13E5), t-HER2, p-Akt, t-Akt, E-cadherin, Vimentin, TCF8/ZEB1, and GAPDH, (1:1,000 dilution; Cell Signaling Technology)
p-Erk1/2 (Thr202/Tyr204), t-ERK1/2, and t-EGFR (1:1000 dilution; R&D systems)
vimentin (ACR 048 A, C; Biocare Medical, Concord, CA, USA)
E-cadherin (M3612; Dako, Santa Clara, CA, USA)
ZEB1 (ab180905; Abcam, Cambridge, UK).

Eukaryotic cell lines

Policy information about cell lines

Cell line source(s)	H2228 (EML4-ALK variant 3a/b E6; A20) cells were purchased from the American Type Culture Collection (Manassas, VA, USA). A925L (EML4-ALK variant 5a, E2: A20) was established from a surgical specimen of the EML4-ALK-positive NSCLC patient and kindly provided by Fumihiro Tanaka of the Second Department of Surgery, University of Occupational and Environmental Health, Japan. The H3122 human lung adenocarcinoma cell line, with EML4-ALK fusion protein variant1 (E13;A20), was kindly provided by Dr. Jeffrey A. Engelman of the Massachusetts General Hospital Cancer Center (Boston, MA). The EML4-ALK-positive NSCLC patient-derived cell lines JFCR-018-1, JFCR-028-3, and JFCR-098 were established from the EML4-ALK-positive NSCLC patient in Hospital of Japanese Foundation for Cancer Research, Japan.
Authentication	Cell lines were authenticated by DNA fingerprinting.
Mycoplasma contamination	Cells were regularly screened for mycoplasma using a MycoAlert Mycoplasma Detection Kit.
Commonly misidentified lines (See ICLAC register)	Commonly misidentified lines were not used in the study.

Animals and other organisms

Policy information about studies involving animals; ARRIVE guidelines recommended for reporting animal research

Laboratory animals	Five-week-old male C.B-17/Icr-scid/scidJcl mice with severe combined immunodeficiency were obtained from Clea Japan (Tokyo, Japan).
Wild animals	The study did not include wild animals.
Field-collected samples	The study did not involve samples collected from the field.
Ethics oversight	Mouse experimental protocols were approved by the institutional review board of Kyoto Prefectural University of Medicine (Kyoto, Japan; approval no. M29-529).

Note that full information on the approval of the study protocol must also be provided in the manuscript.

Human research participants

Policy information about studies involving human research participants

Population characteristics	ALK-rearranged tumor specimens were obtained from 34 patients with NSCLC at University Hospital, Kyoto Prefectural University of Medicine (Kyoto, Japan), Japanese Red Cross Kyoto Daiichi Hospital (Kyoto, Japan), Japanese Red Cross Kyoto Daini Hospital (Kyoto, Japan), and Niigata University Hospital (Niigata, Japan) prior to alectinib treatment.
Recruitment	All patients were participants in Institutional Review Board of each Hospitals –approved studies.
Ethics oversight	All patients were participants in Institutional Review Board of each Hospital –approved studies and all provided written informed consent.

Note that full information on the approval of the study protocol must also be provided in the manuscript.

Flow Cytometry

Plots

Confirm that:

☒ The axis labels state the marker and fluorochrome used (e.g. CD4-FITC).

☒ The axis scales are clearly visible. Include numbers along axes only for bottom left plot of group (a 'group' is an analysis of identical markers).

☐ All plots are contour plots with outliers or pseudocolor plots.

☒ A numerical value for number of cells or percentage (with statistics) is provided.

Methodology

Sample preparation	Cells were collected via centrifugation and resuspended at 1 × 106 cells/mL in propidium iodide (PI) staining buffer (0.1% Triton X-100 and 50 µg/mL PI in PBS)
Instrument	Cell-cycle histograms were generated following the analysis of PI-stained cells by FACS using the BD Accuri™ C6 Plus Flow Cytometer (Becton, Dickinson & Company, Franklin Lakes, NJ).
Software	Histograms generated using FACS were analyzed using FlowJo® V10.6.1 (FlowJo LLC, Ashland, OR) to determine the percentage of cells in each phase (G1, S, and G2–M).
Cell population abundance	For each culture, at least 1 × 104 events were recorded.
Gating strategy	Histograms generated using FACS were analyzed using FlowJo® V10.6.1 (FlowJo LLC, Ashland, OR) to determine the percentage of cells in each phase (G1, S, and G2–M).

☐ Tick this box to confirm that a figure exemplifying the gating strategy is provided in the Supplementary Information.
