# Supplementary material for: HER3 activation contributes toward the emergence of ALK inhibitor-tolerant cells in ALK-rearranged lung cancer with mesenchymal features
Source: NPJ Precis Oncol. 2022 Jan 18;6:5. doi: 10.1038/s41698-021-00250-8 (PMC8766605; doi:10.1038/s41698-021-00250-8)
Supplement: Supplementary file 2 — Sup-Figures [file 41698_2021_250_MOESM2_ESM.pdf]

Supplementary Figure 1

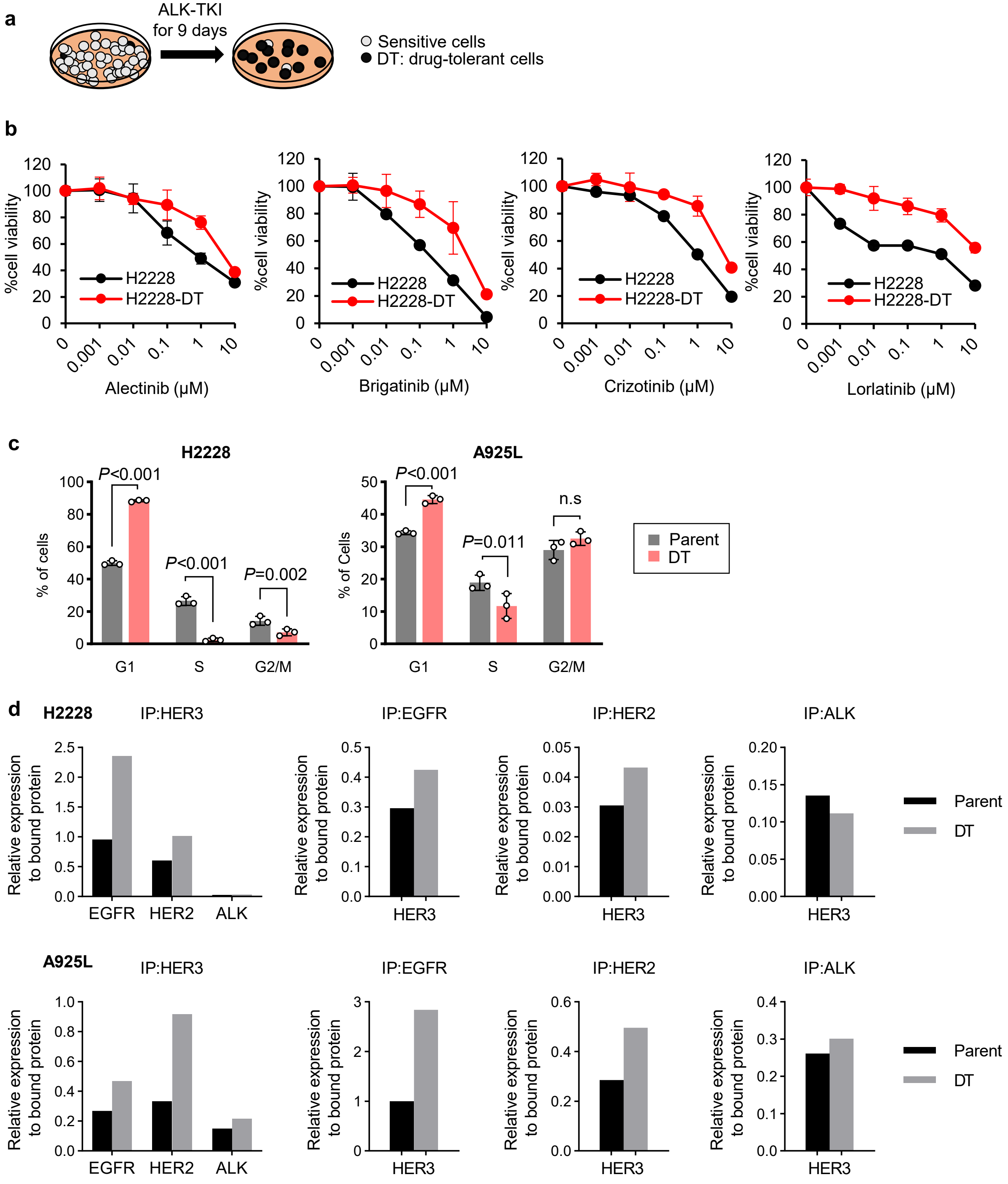

Supplementary Figure 1. G1 arrest and HER family receptor dimerization were associated with drug tolerance in ALK-rearranged NSCLC cells. (a) Plated parental (left) and DT cells (right). DT cells were generated using alectinib (3 μmol/L) or brigatinib (1 μmol/L) for 9 days. **(b)** Cell viability MTT assays of H2228 parental and DT cells incubated with the indicated concentrations of ALK-TKIs (alectinib, brigatinib, crizotinib, or lorlatinib) for 72 h. **(c)** Cell cycle analysis of ALK-rearranged NSCLC cells using flow cytometry and propidium iodide (PI); H2228 and A925L cells were treated with medium or alectinib (3 μmol/L) for 3 days. Results are shown as the percentage of control and alectinib-treated cell populations in various cell cycle phases. $n \geq 2$ independent experiments. *P* values were calculated using two-way ANOVA followed by Tukey's test. Data are represented as mean ± S.D. **(d)** Relative quantification of protein expression bound to indicated proteins in parental and DT cells. The intensities of band in the western blotting with immunoprecipitation were quantified with the ImageLab software version 6.0.1 (Bio-Rad Laboratories, Hercules, CA).

Supplementary Figure 2

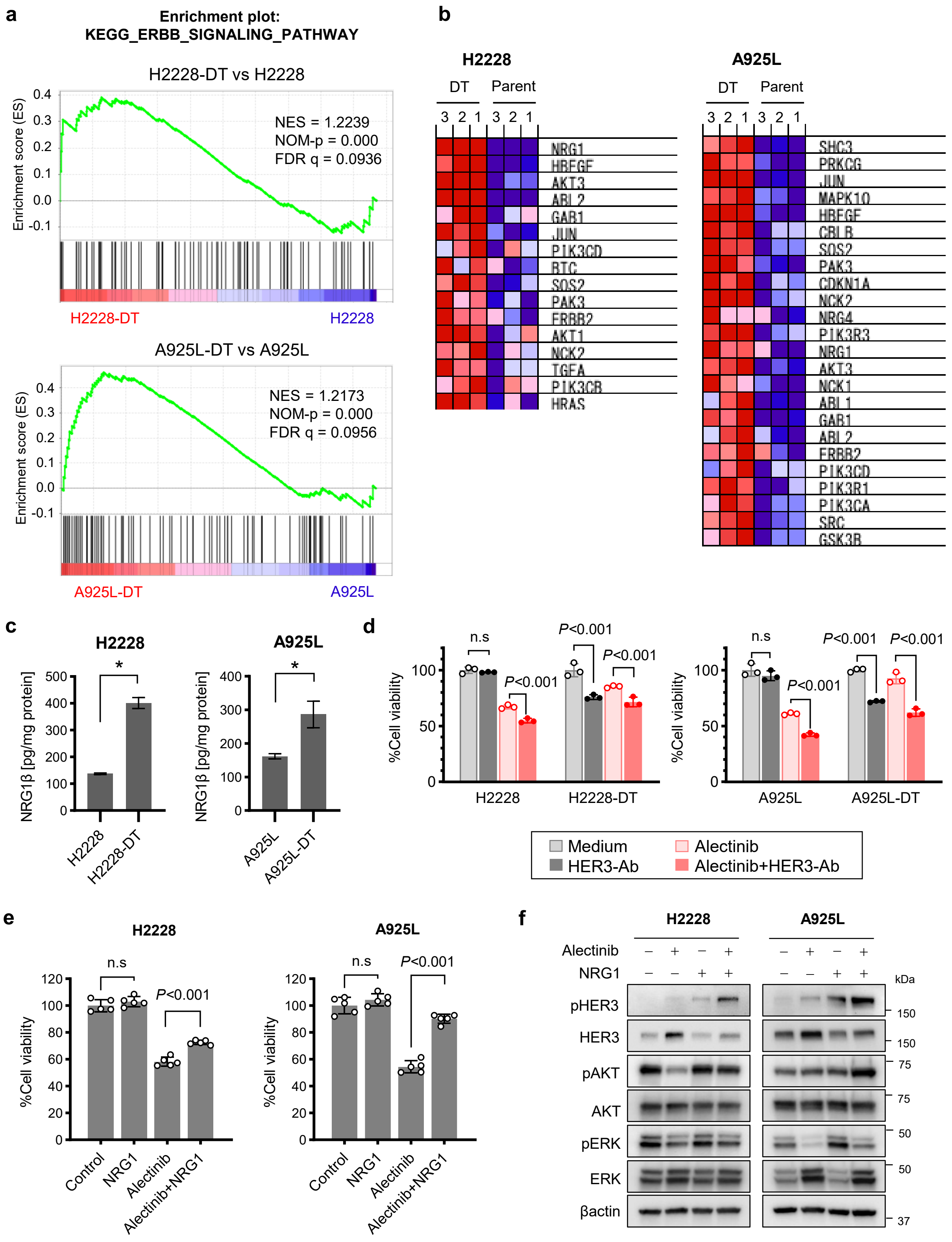

Supplementary Figure 2. HER3 signaling drives drug-tolerance in ALK-rearranged NSCLC cells and their DT cells showed reversibility under the drug free condition *in vitro*. (a) Gene set enrichment analysis (GSEA) from microarray data. Enrichment plot of ERBB_signaling_pathway genes for parental cells vs alectinib-DT H2228 and A925L cells. **(b)** The corresponding heat map of the core enrichment genes. **(c)** NRG1β protein production in the lysate of H2228 and A925L parental and DT cells were determined by ELISA. **(d)** Cell viability MTT assay results for H2228 and A925L parental and DT cells incubated with medium only, alectinib (100 nmol/L), anti-HER3 antibodies (3 μg/mL), or a combination for 72 h. **(e)** MTT assays of H2228 and A925L parental cells incubated with medium only, alectinib (100 nmol/L), NRG1 (100 ng/mL), or a combination for 72 h. **(f)** Western blotting analysis of H2228 and A925L parental cells incubated with medium only, alectinib (100 nmol/L), NRG1 (100 ng/mL), or a combination for 24 h. n ≥ 2 independent experiments. *$P < 0.5$ (c: unpaired *t*-test, d,e: two-way ANOVA). Data are represented as mean ± S.D.

Supplementary Figure 3

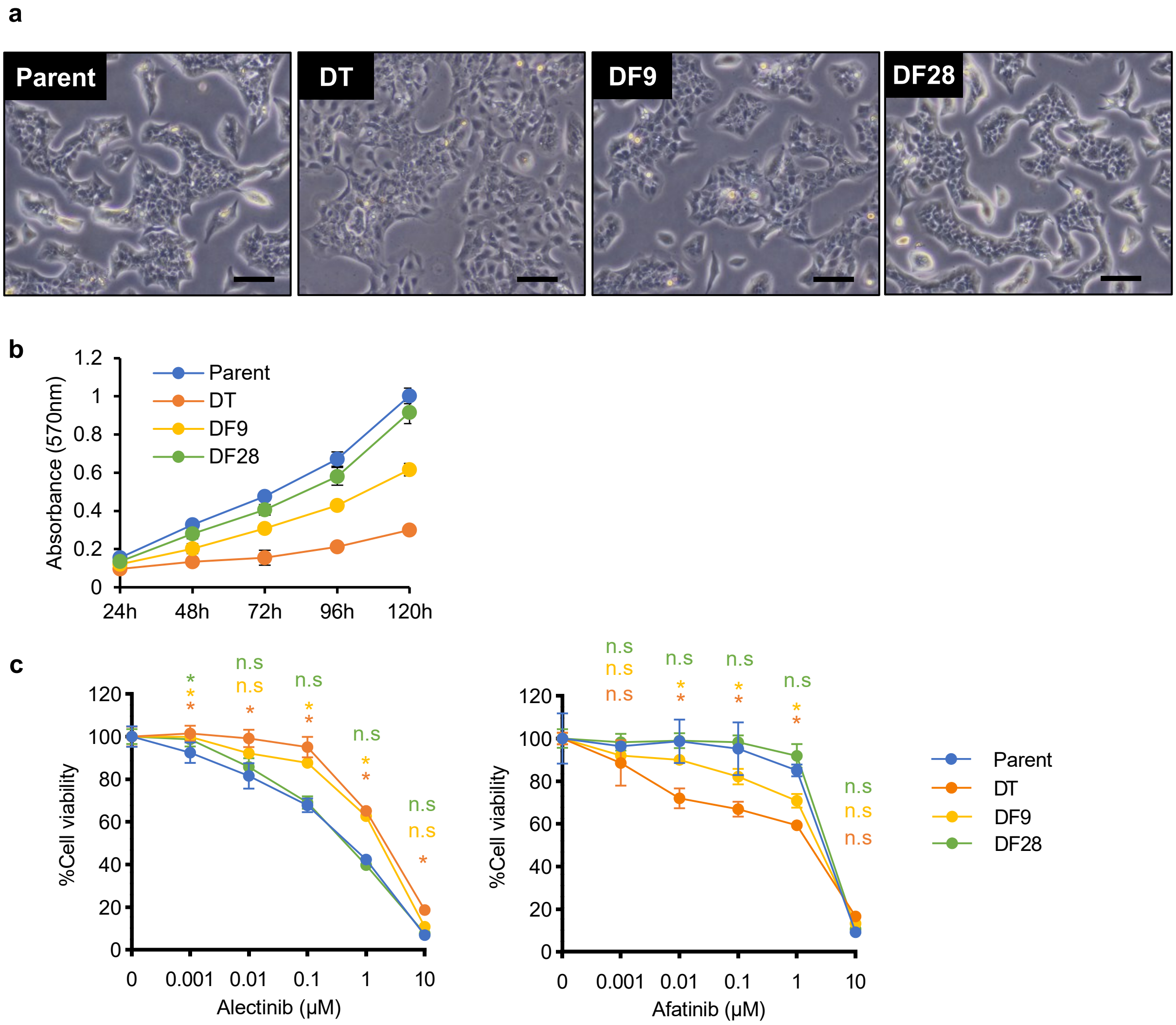

Supplementary Figure 3. DT cells in ALK-rearranged NSCLC cells showed reversibility under the drug free condition *in vitro*. (a-c) Plated H2228 cells were untreated or treated with alectinib (3 μmol/L) for 9 days to establish DT cells, which were incubated in drug-free medium for 9 days (DF9) or 28 days (DF28). **(a)** Cells were evaluated using a light microscope to determine morphological changes. Scale bar, 100 μm. **(b)** Cell viability MTT assay results of cells incubated for the indicated durations. **(c)** MTT assay results of cells incubated with the indicated concentrations of alectinib or afatinib for 72 h. $^{*}P < 0.05$ vs parental cells (two-way ANOVA followed by Dunnett's test). Data are represented as mean ± S.D.

Supplementary Figure 4

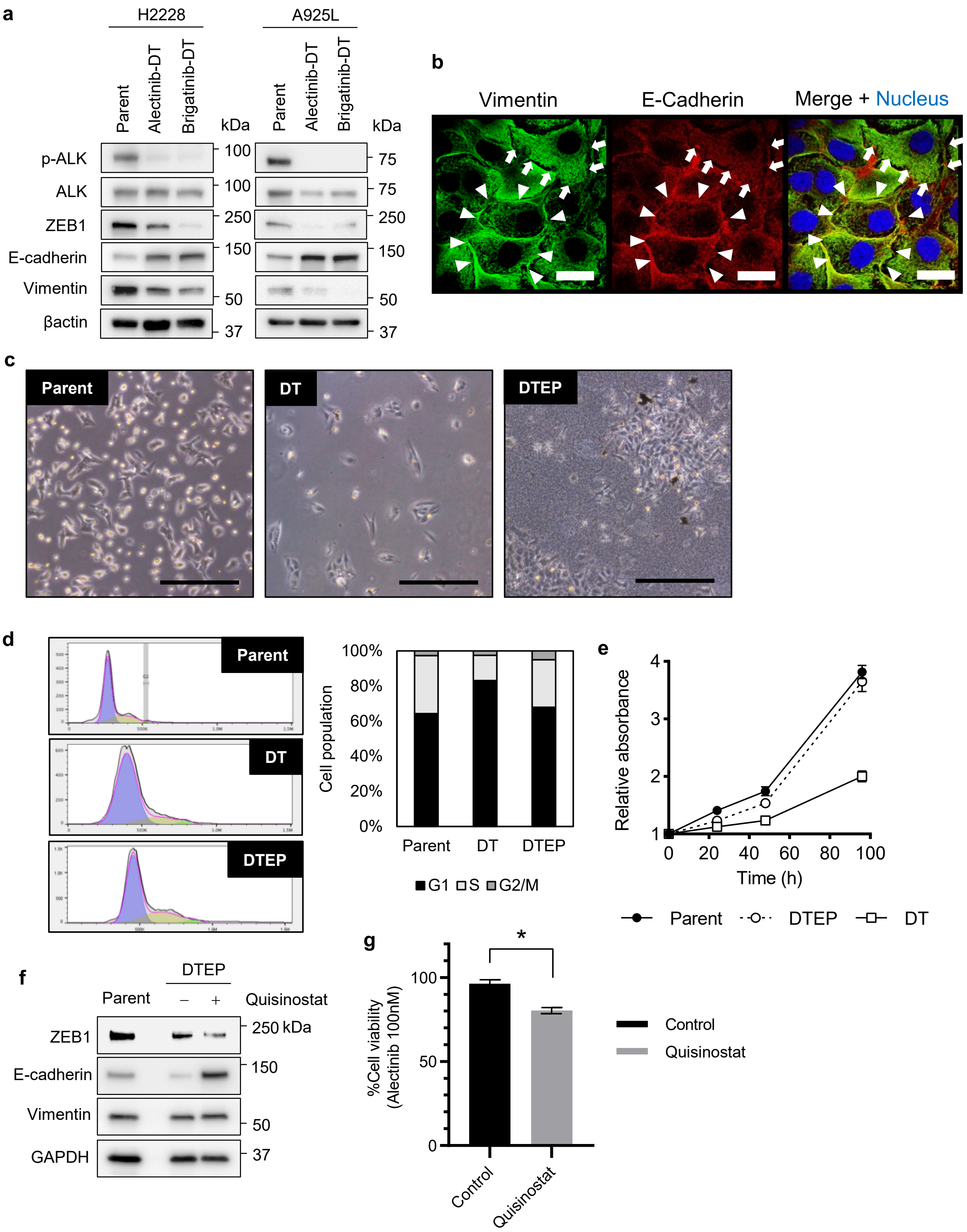

Supplementary Figure 4. The effect of ALK-TKI on alterations in EMT marker expression in ALK-rearranged NSCLC cells and the impact of drug-tolerant expanded persisters. (a) Western blotting results of H2228 and A925L parental and DT cells. **(b)** Immunocytochemical analysis of H2228 cells treated with alectinib (3 μmol/L) for 96 h. Arrowheads indicate E-cadherin expression and arrows indicate vimentin expression. **(c-e)** Plated H2228 cells were untreated or treated with alectinib (3 μmol/L) for 9 days to establish drug-tolerant (DT) cells, or for 30 days to establish drug-tolerant expanded persister (DTEP) cells. Scale bar, 500 μm. **(c)** Cells were evaluated using a light microscope to determine morphological changes. Scale bar, 500 μm. **(d)** Cell cycle analysis of H2228 parental, DT, and DTEP cells treated with medium or alectinib (3 μmol/L) for 9 days and 30 days using flow cytometry with PI. Results are shown as the percentage of control and alectinib-treated cell populations in various cell cycle phases; $n \geq 3$ independent experiments. **(e)** MTT assays of H2228 parental, DT, and DTEP cells incubated for the indicated times. *$P < 0.05$ vs parent cells (two-way ANOVA followed by Dunnett's test). **(f)** Western blotting results of H2228 parental and DTEP cells which were treated or untreated with quisinostat (30nmol/L) for 72 h. **(g)** Cell viability MTT assay results of DTEP cells, which were pretreated or untreated with quisinostat for 72h, incubated with 100 nmol/L of alectinib for 72h. *$P < 0.5$ (unpaired *t*-test). Data are represented as mean ± S.D.

Supplementary Figure 5

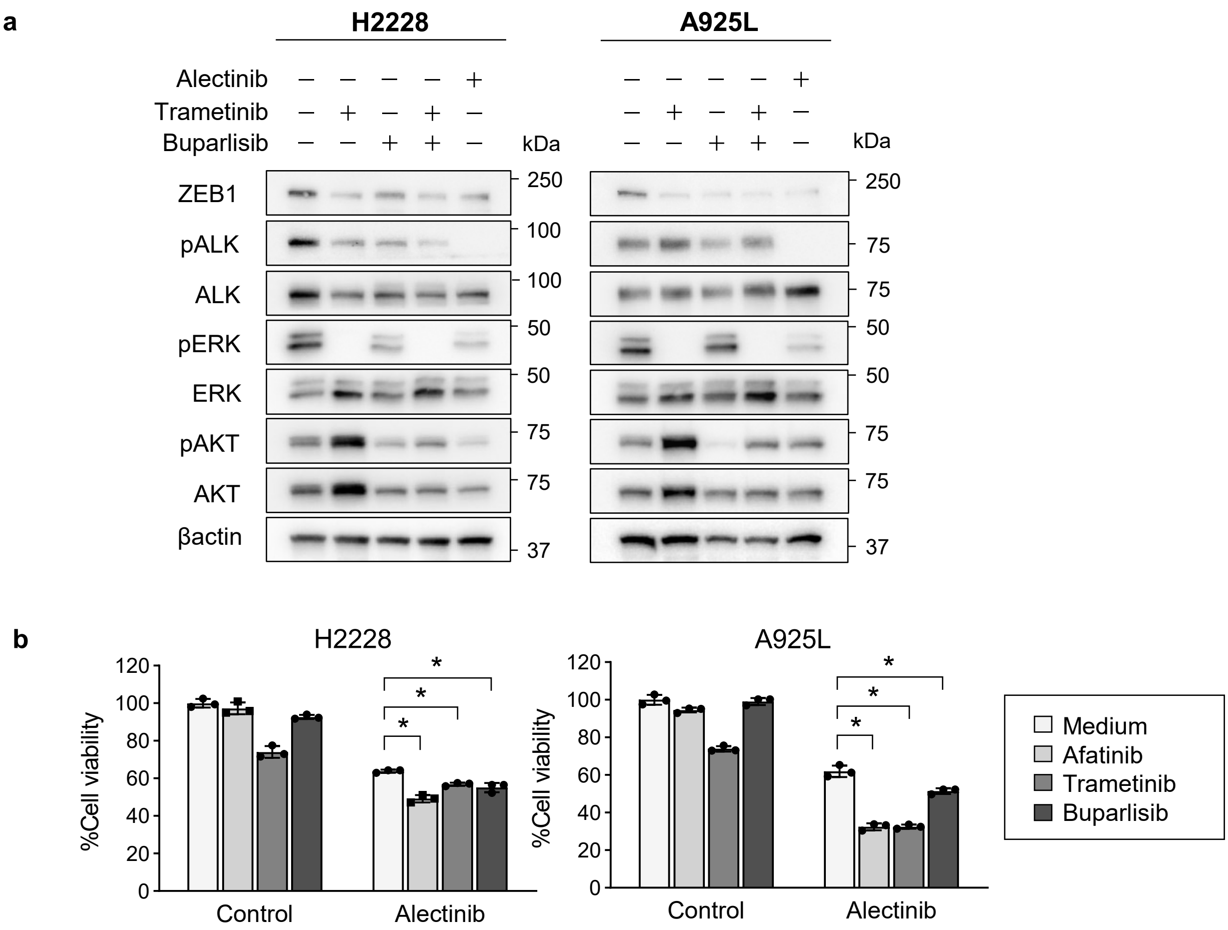

Supplementary Figure 5. The effect of ALK, HER3, MEK, or PI3K-AKT inhibitors on the ZEB1 protein expression level and the viability of ALK-rearranged NSCLC cells. **(a)** Western blotting results of H2228 and A925L parental cells incubated with medium only, trametinib (100 nmol/L), and buparlisib (100 nmol/L), or alectinib (100 nmol/L) for 24 h. **(b)** Cell viability MTT assay results of H2228 and A925L cells incubated with medium only, afatinib (100 nmol/L), trametinib (100 nmol/L), and buparlisib (100 nmol/L), with or without alectinib (100 nmol/L), for 72 h. **P* < 0.05 vs alectinib monotherapy (one-way ANOVA followed by Dunnett's test). Data are represented as mean ± S.D.

Supplementary Figure 6

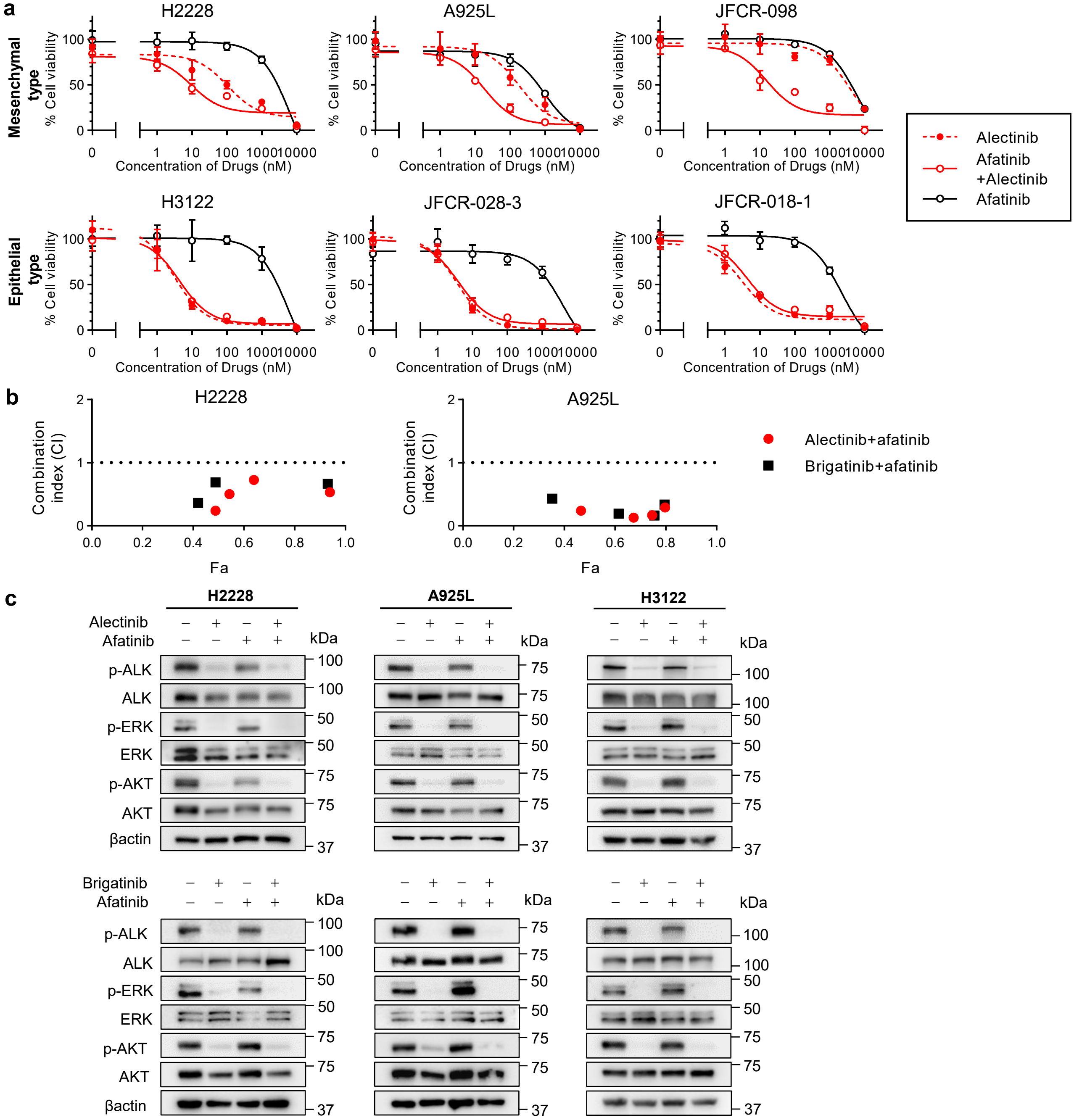

Supplementary Figure 6. The effect of cotreatment with ALK-TKI and afatinib on the viability of ALK-rearranged NSCLC cells *in vitro*. (a) MTT assay results of five ALK-rearranged NSCLC cell lines incubated with the indicated concentrations of alectinib, afatinib, or a combination of alectinib plus afatinib (100 nmol/L) for 72 h. Data are represented as mean ± S.D. **(b)** Combination index (CI) values were analyzed according to the Chou and Talalay equation using the CalcuSyn software. **(c)** Western blotting results of H2228, A925L, or H3122 parental cells incubated with medium only, alectinib (100 nmol/L), brigatinib (100 nmol/L), afatinib (100 nmol/L), or a combination for 4 h.

Supplementary Figure 7

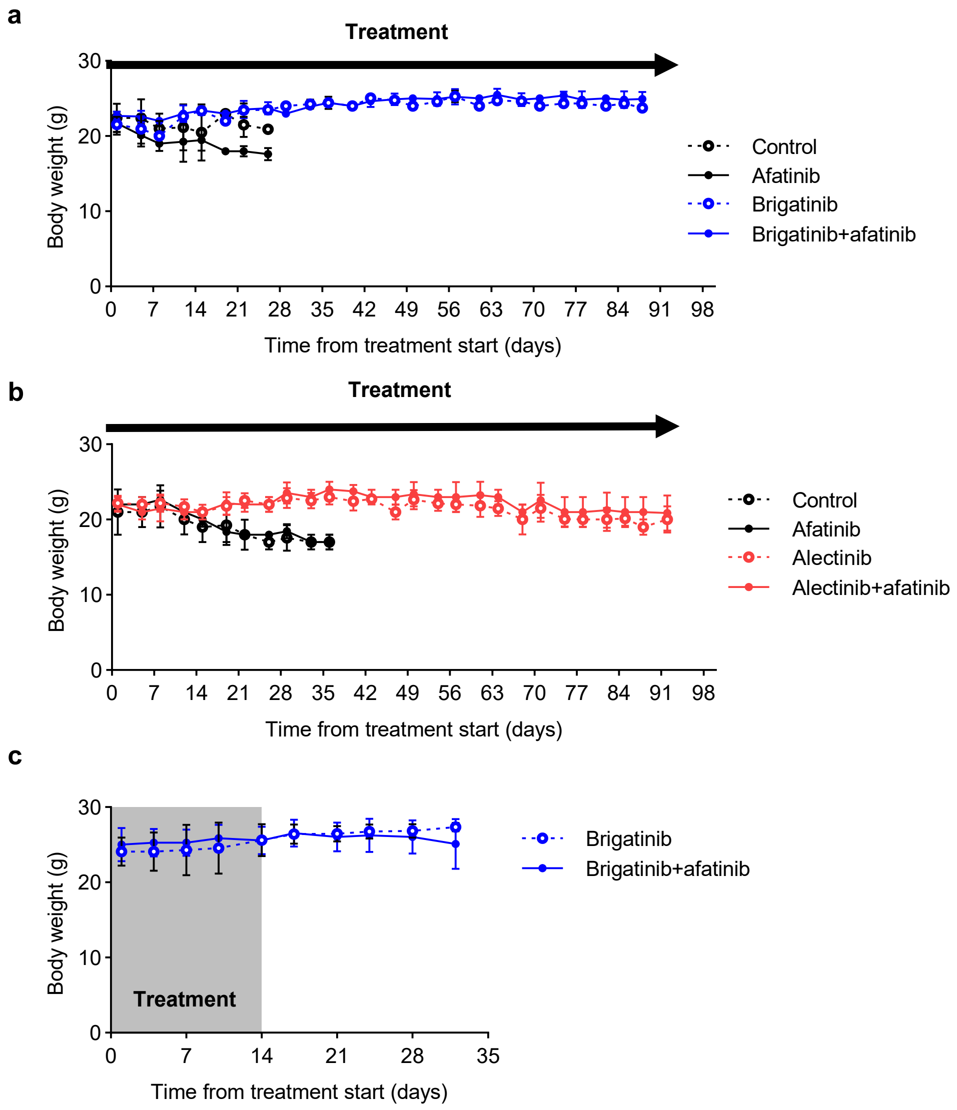

Supplementary Figure 7. Therapeutic tolerance in xenograft models of human ALK-rearranged NSCLC. Mouse weight was evaluated twice weekly. **(a)** A925L CDX tumors were treated with a vehicle (control), brigatinib (10 mg/kg), afatinib (5 mg/kg), or brigatinib (10 mg/kg) plus afatinib (5mg/kg) (n = 6) via daily oral gavage. **(b)** H2228 CDX tumors were treated with a vehicle (control), alectinib (6 mg/kg), afatinib (5 mg/kg), or alectinib (6 mg/kg) plus afatinib (5 mg/kg) (n = 6) via daily oral gavage. **(c)** A925L CDX tumors were treated with brigatinib (10 mg/kg) or brigatinib (10 mg/kg) plus afatinib (5 mg/kg) (n = 5) via daily oral gavage. Data are represented as mean ± S.D.

Supplementary Figure 8

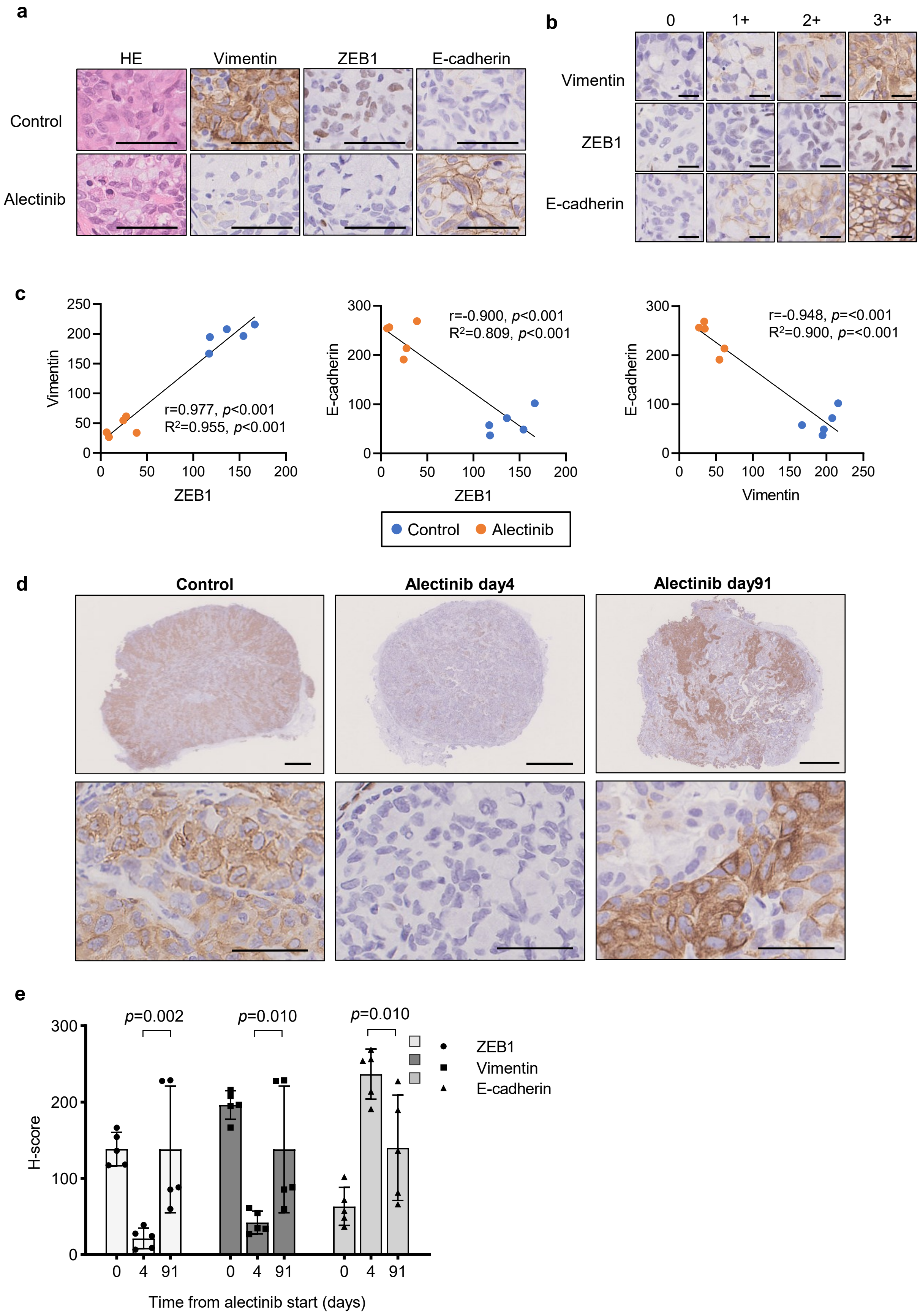

Supplementary Figure 8. Alternation of EMT marker expressions with drug treatment in xenograft models of ALK-rearranged NSCLC cells. (a) Representative immunohistochemistry staining for vimentin and E-cadherin expression in H2228 CDX tumors treated with or without alectinib for 4 days. Scale bar, 50 µm. **(b)** Vimentin and E-cadherin intensity scoring. H-scores were calculated from the percentage of cells with each score. Scale bar, 20 µm. **(c)** Correlation between vimentin, E-cadherin, and ZEB1 expression in lysates from H2228 CDX tumors. *P* values were calculated using Pearson's correlation coefficient and linear regression analysis. **(d)** Representative immunohistochemistry staining of vimentin expression in H2228 CDX tumors treated with a vehicle (control) or alectinib for 4 or 91 days. Scale bar, 1 mm (upper panel) and 50 µm (lower panel). **(e)** Quantification of immunostaining-positive cells, as determined by the H-score for vimentin, ZEB1, and E-cadherin calculated from areas randomly selected from CDX tumors treated with vehicle or alectinib (6 mg/kg) for 4 or 91 days. *P* values were calculated using two-way ANOVA followed by Tukey's test. Data are represented as mean ± S.D.

Supplementary Figure 9

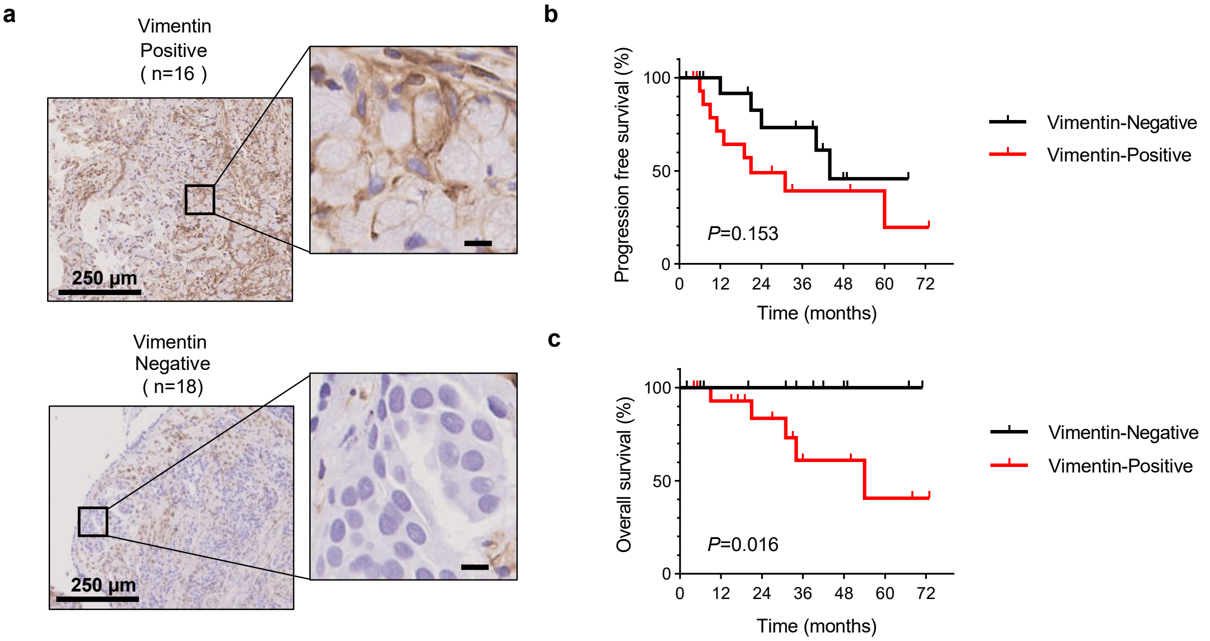

Supplementary Figure 9. Clinical significance of vimentin expression in the outcome of alectinib treatment in patients with ALK-rearranged NSCLC. (a) Representative immunohistochemistry images of clinical specimens stained with human-specific vimentin antibodies. Scale bar, 10 μm. **(b)** Kaplan-Meier analysis of progression-free survival following alectinib treatment. **(c)** Kaplan-Meier analysis of overall survival following alectinib treatment. HR < 1 indicates a low risk of progression in the vimentin-expression group: **(b)** HR 2.15 (95% CI: 0.75-6.35, $P = 0.153$) and **(c)** HR 8.70 (95% CI: 1.49-50.86, $P = 0.016$).

	Vimentin-positive (n = 16)	Vimentin-negative (n = 18)
**Characteristics**		
Age-median (range), years	67 (42-85)	65.5 (53-81)
Sex-n.		
Male	9	8
Female	7	10
Smoking history-n.		
Current or former	9	9
Never	7	9
Histology-n.		
Adenocarcinoma	16	18
Other	0	0
Clinical stage -n.		
III A-III C	3	5
IV A	3	6
IV B	6	2
Postoperative recurrence	4	5

Supplementary Table 1: Patients characteristics

Supplementary Methods

Flow cytometry

Cells were collected via centrifugation and resuspended at 1 × 10^6 cells/mL in propidium iodide (PI) staining buffer (0.1% Triton X-100 and 50 μg/mL PI in PBS). Cell-cycle histograms were generated following the analysis of PI-stained cells by FACS using the BD Accuri™ C6 Plus Flow Cytometer (Becton, Dickinson & Company, Franklin Lakes, NJ). For each culture, at least 1 × 10^4 events were recorded. Histograms generated using FACS were analyzed using FlowJo® V10.6.1 (FlowJo LLC, Ashland, OR) to determine the percentage of cells in each phase (G1, S, and G2–M).

Cytokine production

Parental and DT cells derived from H2228 and A925L cells (2 × 10^6) were lysed, and the lysates were stored at -70 °C until analysis. Level of NRG1β was determined with Human NRG1β DuoSet ELISA kit (R&D Systems), according to the manufacturer's protocols. All lysates were tested twice. Color intensity was measured at 450 nm using a spectrophotometric plate reader. Concentrations of growth factors were determined from standard curves. The amount of protein was measured by the bicinchoninic acid assay and standardized the sample protein.

Drug combination studies

Synergistic interactions were quantified through the isobologram and combination index methods proposed by Chou and Talalay [S1] using the CalcuSyn software (Biosoft). The combination index (CI) is a quantitative representation of two-drug pharmacologic interactions. A CI of 1 indicates additivity between the two agents, whereas a CI < 1 or CI > 1 indicates synergism or antagonism, respectively.

Supplementary Reference

S1. Chou TC&Talalay P. Quantitative analysis of dose-effect relationships: the combined effects of multiple drugs or enzyme inhibitors. *dv Enzyme Regul* **22**: 27-55 (1984).

Full blots images

Fig.1c

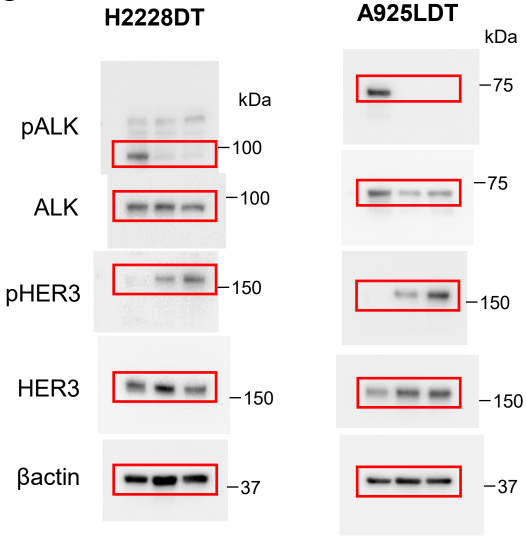

Fig.1d

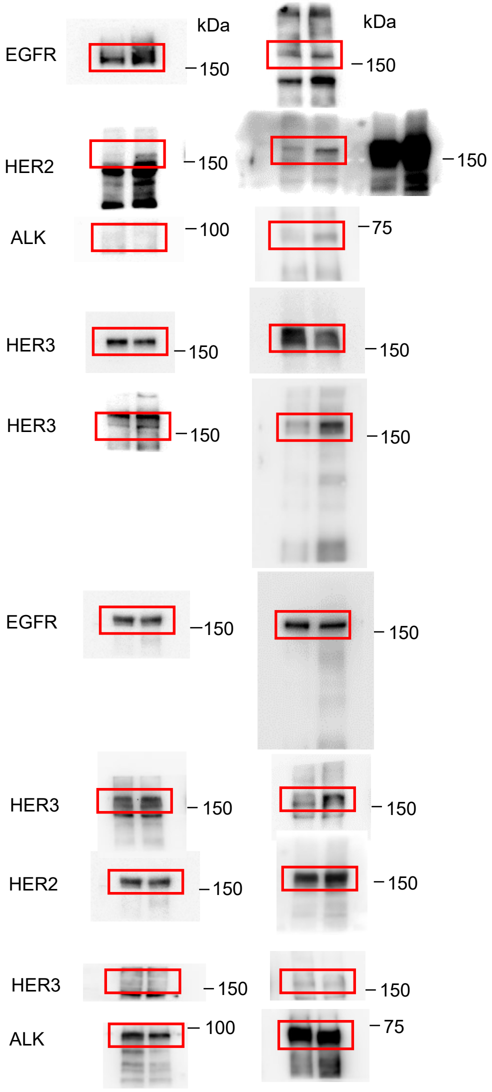

Fig.1j

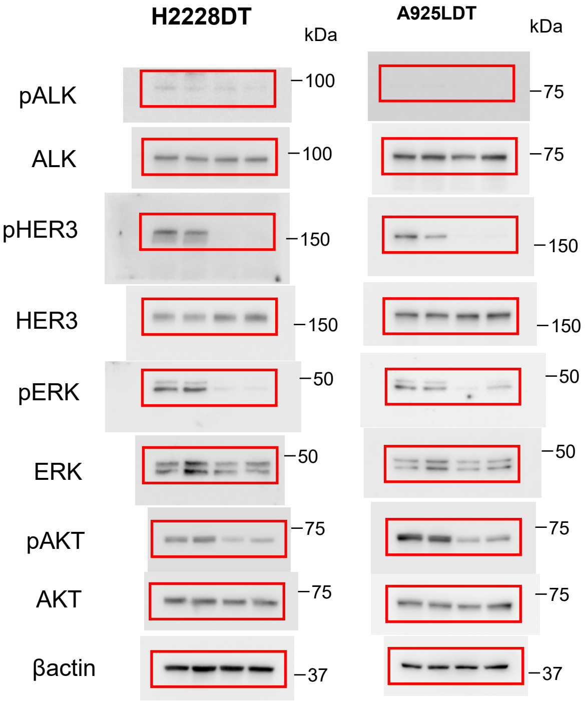

Fig.2b

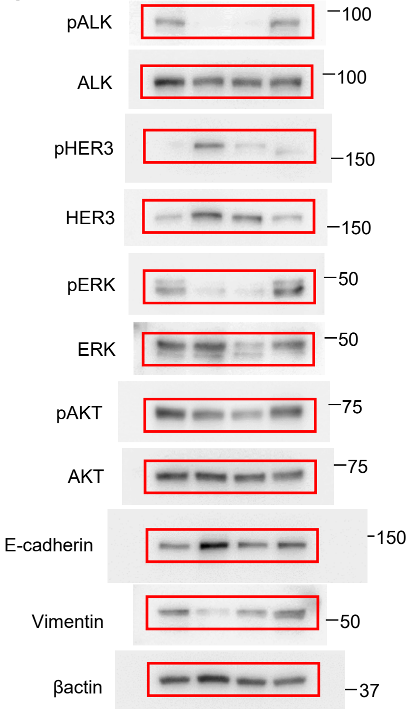

Fig.2d

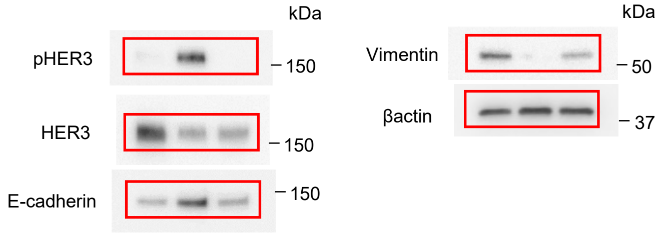

Fig.2g

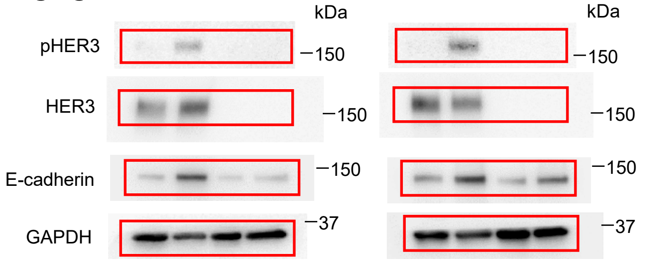

Fig.2j

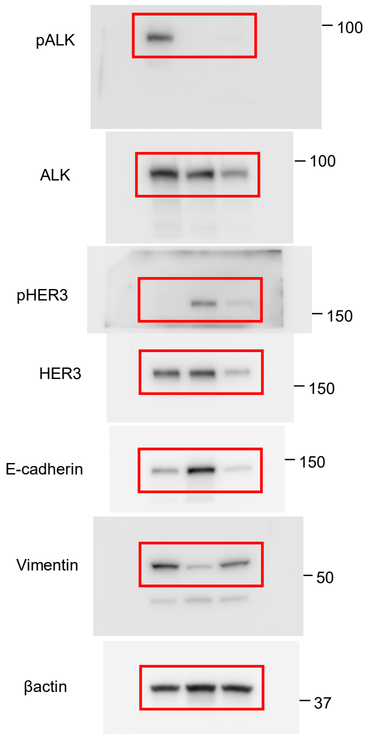

Fig.3a

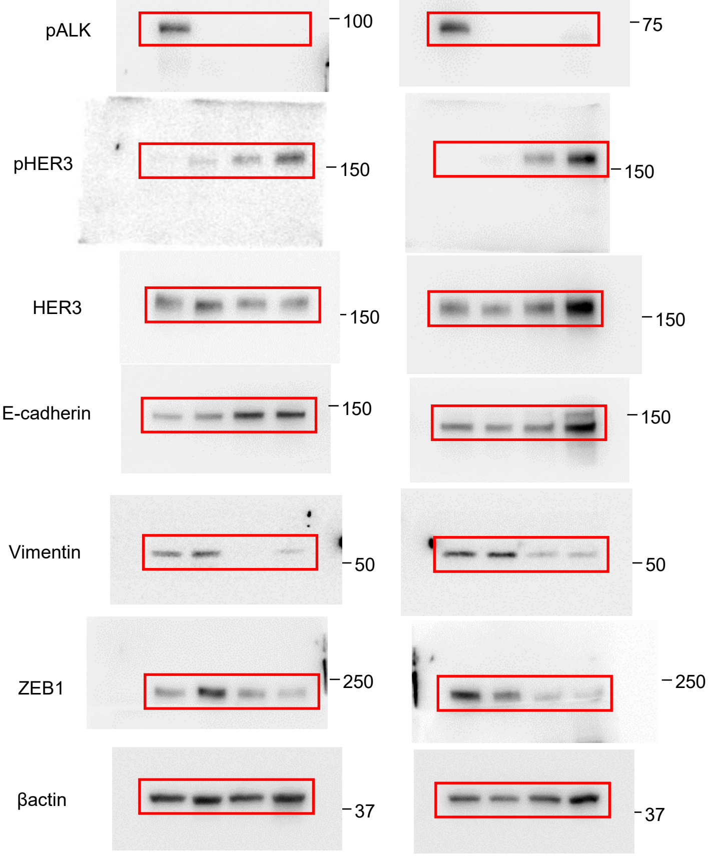

Fig.3b

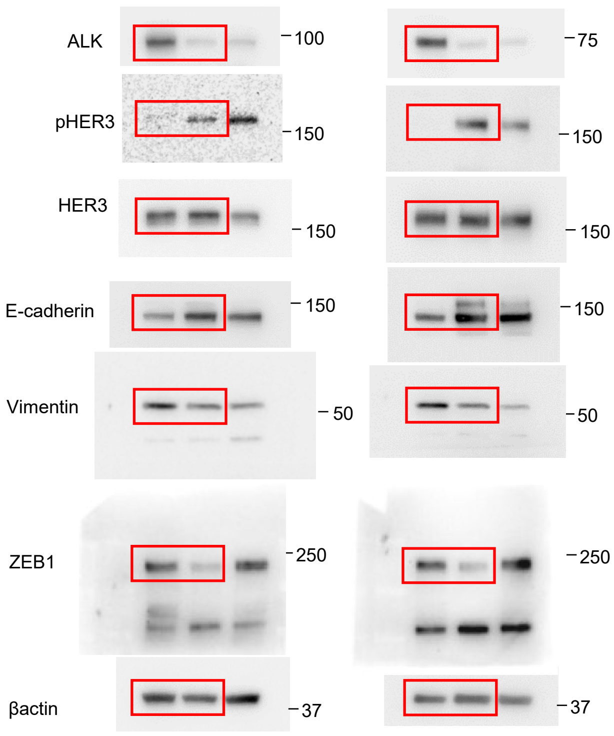

Fig.3c

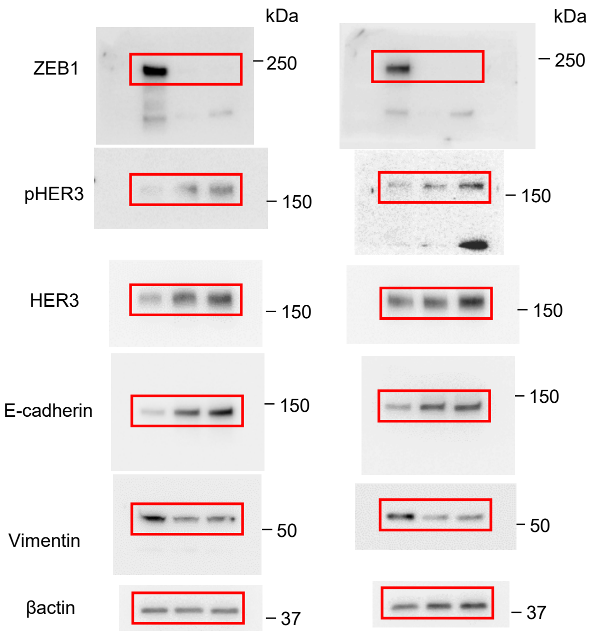

Fig.3d

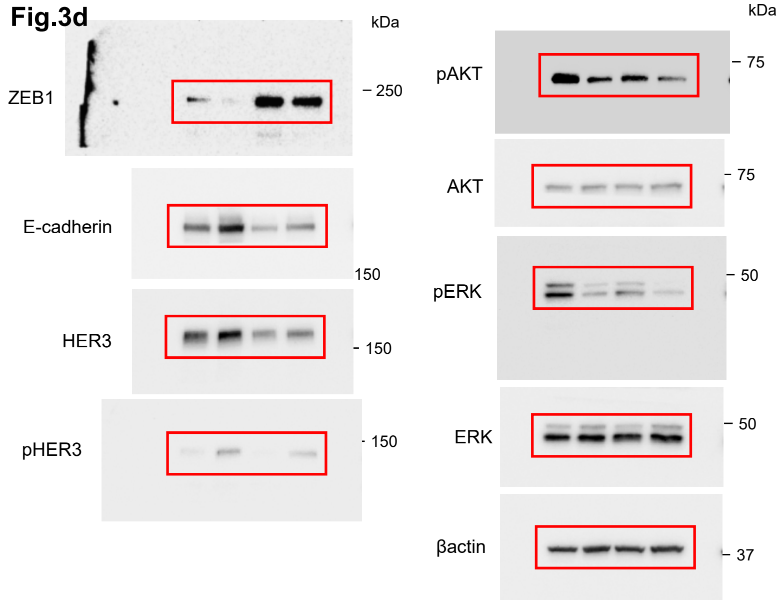

Fig.4a

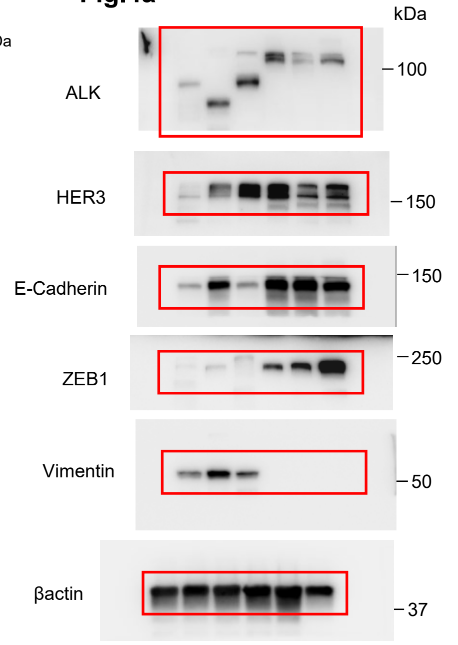

Fig.4e

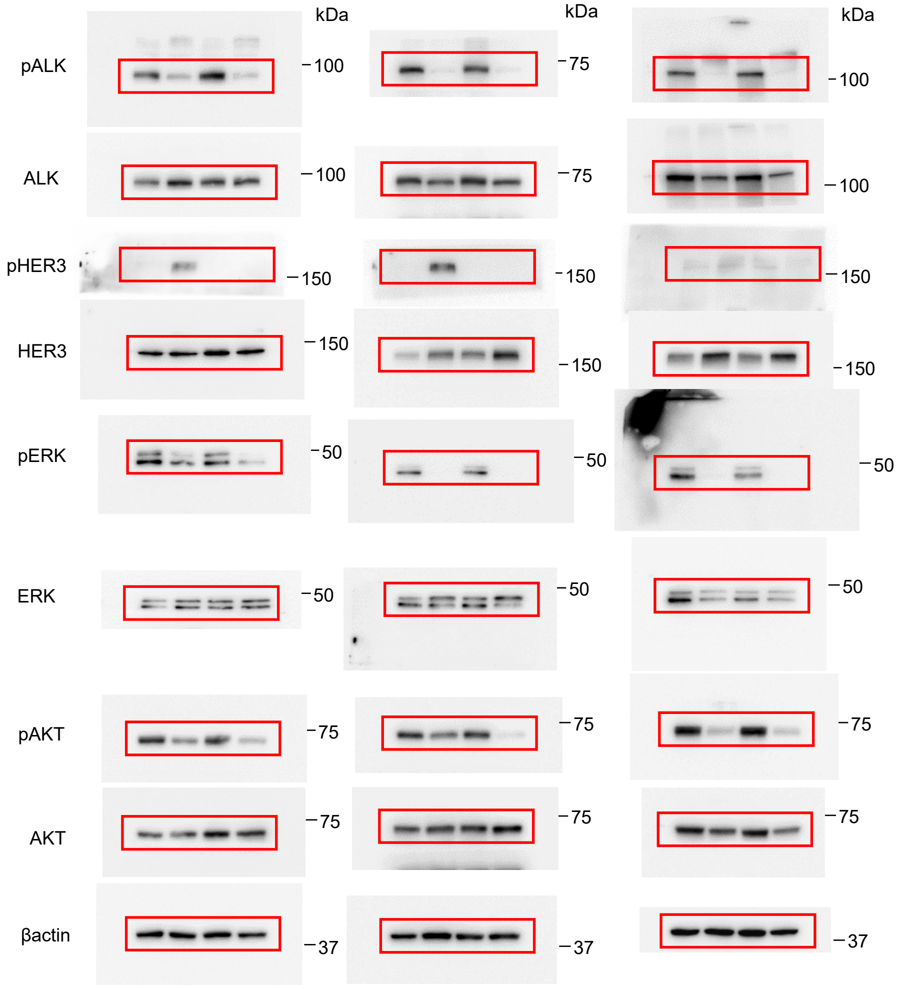
kDa
pALK
100
75
ALK
pHER3
150
HER3
pERK
50
ERK
pAKT
AKT
βactin
37
